# Unveiling a Hidden Conversion Pathway in CoSe_2_ Anodes via Rationally Designed CNT‐Interwoven Hollow Carbon Microclusters for High‐Performance Potassium‐Ion Batteries

**DOI:** 10.1002/advs.76162

**Published:** 2026-06-22

**Authors:** Ho Rim Kim, Seohyeon Jang, Hong Geun Oh, Jaewoo Lee, Jihun Yeom, Daiha Shin, Jiung Cho, Inho Nam, Seung‐Keun Park

**Affiliations:** ^1^ Department of Intelligent Energy and Industry Chung‐Ang University Seoul Republic of Korea; ^2^ Metropolitan Seoul Center Korea Basic Science Institute (KBSI) Seoul Republic of Korea; ^3^ Department of Materials Science and Engineering Hongik University Sejong Republic of Korea; ^4^ Department of Chemical Engineering Chung‐Ang University Seoul Republic of Korea; ^5^ Department of Advanced Materials Engineering Chung‐Ang University Gyeonggi‐do Republic of Korea

**Keywords:** carbon nanotubes, cobalt selenides, hollow carbon sphere, K‐ion storage mechanism, potassium‐ion battery, spray drying

## Abstract

The search for sustainable energy storage has positioned potassium‐ion batteries (PIBs) as a compelling alternative to lithium‐ion systems, yet the lack of robust anode materials remains a significant hurdle. Transition‐metal selenides, particularly CoSe_2_, are attractive for their high theoretical capacity and fast kinetics, but they inevitably succumb to catastrophic structural failure. This degradation is rooted in the traditional stepwise insertion pathway (CoSe_2_ → K_x_CoSe_2_ → Co + K_2_Se), which triggers a relentless lattice expansion exceeding 250%. Herein, we report the rational design of CNT‐interwoven hollow carbon microclusters as a rigid nanoconfined host to modulate this reaction behavior. This specific architecture does more than buffer volume changes; it fundamentally steers the electrochemical behavior toward a previously hidden conversion‐insertion mechanism mediated by a Co_3_Se_4_ intermediate. Thermodynamic analysis via first‐principles calculations confirms that this newly unearthed route effectively sequesters major strain during the initial CoSe_2_ → Co_3_Se_4_ transition, thereby preserving the electrode's integrity throughout subsequent potassiation. In situ X‐ray diffraction provides direct, real‐time evidence of this stabilized Co_3_Se_4_ phase and its smooth evolution into K_x_Co_3_Se_4_, bypassing the destructive traditional landscape. Our findings underscore that meticulous structural tailoring can be a powerful tool to capture elusive intermediates and redefine reaction pathways for high‐endurance PIB anodes.

## Graphical abstract

A rational architectural design of hierarchical, CNT‐interwoven hollow carbon microclusters unlocks and stabilizes a unique monoclinic Co_3_Se_4_‐mediated conversion–insertion pathway for potassium storage. This structural confinement effectively guides the reaction kinetics and accommodates severe mechanical strain. Consequently, the engineered anode ensures exceptional phase reversibility and structural integrity, offering breakthrough insights into conversion‐type battery chemistry.

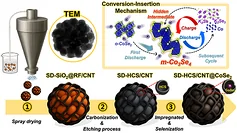

## 1 Introduction

Potassium‐ion batteries (PIBs) have emerged as promising alternatives to lithium‐ion systems due to the natural abundance and low cost of potassium resources [1, 2, 3, 4, 5, 6, 7, 8, 9, 10, 11, 12]. In principle, PIBs can provide comparable energy density at lower cost, but the large ionic radius of K^+^ (1.38 Å compared with 0.76 Å for Li^+^) introduces severe strain on the host materials, hindering the development of suitable anodes [13, 14, 15, 16].

Transition‐metal selenides (TMSe) have emerged as promising PIB anodes owing to their high capacity and favorable conversion kinetics enabled by relatively weak M─Se bonds [17, 18, 19, 20, 21, 22]. Among them, CoSe_2_ stands out due to its high electronic conductivity and rich redox chemistry, facilitating efficient and reversible electrochemical reactions [23, 24, 25, 26, 27, 28, 29]. However, CoSe_2_ suffers from severe structural degradation during cycling, similar to other conversion‐type electrodes [30, 31]. The conventional mechanism involves the sequential intercalation of K^+^ into CoSe_2_, yielding ternary selenides (CoKSe_2_, CoK_2_Se_2_, CoK_3_Se_2_) before fully converting to metallic Co and K_2_Se [32, 33]. This stepwise insertion pathway induces lattice expansion of over 250% relative to pristine CoSe_2_, far exceeding the mechanical tolerance of typical electrode architectures and leading to catastrophic mechanical strain, electrical network disruption, and capacity fading acceleration. Nanostructuring and carbon‐compositing strategies have been proposed to mitigate these problems; however, the intrinsic instability of the conventional pathway continues to limit performance. This raises the question of whether an alternative mechanistic route may provide a more realistic and sustainable pathway for TMS‐conversion‐type electrodes.

This study reexamined the conventional mechanism from a thermodynamic perspective. According to the Co─Se phase diagram, Co_3_Se_4_ is thermodynamically related to CoSe_2_ within certain compositional and temperature ranges. Therefore, instead of undergoing sequential intercalation through high‐strain ternary intermediates, CoSe_2_ follows a conversion–insertion pathway that first produces Co_3_Se_4_ and K_2_Se, followed by K^+^ insertion to form Co_3_KSe_4_, which ultimately decomposes into metallic Co and potassium selenides (K_2_Se and K_2_Se_3_). Density functional theory (DFT) calculations strongly support this mechanism. The formation energies for conventional stepwise intermediates are highly positive (+1.63, +3.86, +1.65 eV), with only the final step being favorable (−6.29 eV). In contrast, the Co_3_Se_4_‐mediated pathway exhibits consistently negative formation energies (−2.11, −0.26, −1.81 eV), indicating thermodynamic accessibility.

Volume expansion analysis further differentiates these pathways. In the stepwise insertion route, the lattice volumes increase drastically from CoSe_2_ to CoKSe_2_ (+166%), CoK_2_Se_2_ (+259%), and CoK_3_Se_2_ (+267%), undermining long‐term stability. In contrast, the Co_3_Se_4_ pathway localizes the largest volume change at the initial CoSe_2_ → Co_3_Se_4_ transition, which occurs without consuming potassium and concentrates the most destructive strain. Subsequent insertion into Co_3_Se_4_ to form Co_3_KSe_4_ increases the volume only to 141%, indicating that the absence of potassium consumption in the first irreversible step confines the major structural stress to this single transition, whereas the later stages are far more tolerable, suggesting that sustainable cycling may be achieved if the initial transition is buffered.

Despite its thermodynamic favorability, the Co_3_Se_4_‐mediated pathway has rarely been reported [34], possibly owing to the kinetic barriers associated with the first irreversible transition. The rapid and destructive expansion involved in Co_3_Se_4_ formation generates severe strain, suppressing the nucleation of the intermediate. Although Ostwald's rule dictates that intermediates form preferentially, the large lattice mismatch and associated elastic energy barrier between CoSe_2_ and Co_3_Se_4_ hinder the stabilization of Co_3_Se_4_ [35]. Instead, the direct conversion to Co‐ and K‐selenides is often observed. Moreover, extreme strain at the early stage frequently triggers lattice collapse, making strain relaxation a faster kinetic solution compared with intermediate stabilization. Previous studies have occasionally detected transient Co_3_Se_4_ signatures, but only in negligible amounts, leading to their dismissal as a byproduct rather than being recognized as a key mechanistic phase. This highlights the need for electrode designs that maintain Co_3_Se_4_ stability during cycling, aligning experimental observations with thermodynamic predictions.

This study constructed an ideal electrode environment to stabilize the CoSe_2_ → Co_3_Se_4_ pathway and reveal its role in potassium storage, focusing on a carbon nanotube (CNT)‐driven self‐assembly approach to create hierarchical hollow carbon sphere (HCS) microclusters (referred to as SD‐HCS/CNT). The CoSe_2_ nanoparticles are uniformly confined within the hollow carbon framework, reducing the nucleation barriers and restricting uncontrolled growth. The interwoven CNT skeleton provides a robust 3D conductive scaffold that preserves electrical pathways and redistributes stress during cycling. The hollow interior acts as a strain buffer, accommodating large volume fluctuations and mitigating catastrophic collapse during the critical first transition. This rational structural design can realize a thermodynamically preferred Co_3_Se_4_‐mediated pathway.

The hypothesis was validated experimentally. The phase evolution was directly captured through in situ X‐ray diffraction (XRD) measurements performed during potassiation and depotassiation. In SD‐HCS/CNT, the orthorhombic CoSe_2_ reflections diminished upon discharge, and new peaks corresponding to monoclinic Co_3_Se_4_ emerged, providing evidence for the intermediate. Distinct reflections of K‐inserted Co_3_KSe_4_ were also detected, consistent with the proposed pathway. Importantly, Co_3_Se_4_ appeared after the complete disappearance of CoSe_2_, indicating that although conversion begins with CoSe_2_, the subsequent reversible cycling proceeds primarily through Co_3_Se_4_. This confirms that Co_3_Se_4_ is the key active intermediate governing reversibility, consistent with thermodynamic predictions. First‐principles calculations and in situ structural characterization revealed that competition between thermodynamic stability and kinetic strain relaxation is a general rule governing conversion chemistry. The rational electrode design, achieved using SD‐HCS/CNT, can stabilize hidden intermediates, unlocking sustainable cycling behavior. This study redefined the mechanistic landscape of conversion electrodes, offering a blueprint for rationally controlling reaction pathways in diverse energy storage materials.

## 2 Results and Discussion

To investigate the possible reaction pathway of CoSe_2_ during potassiation and identify the step inducing the most severe structural strain, DFT calculations were carried out before experimental validation. According to the Co─Se phase diagram, Co_3_Se_4_ exists as a thermodynamically related phase to CoSe_2_ over certain compositional and temperature ranges, suggesting that partial conversion of CoSe_2_ to Co_3_Se_4_ can occur under electrochemical conditions [36]. Accordingly, various plausible conversion and insertion sequences were considered to determine the most favorable reaction route. To quantitatively compare the different reaction pathways, the overall potassiation process was classified into two distinct routes. The first route is the stepwise K^+^ insertion pathway into CoSe_2_, wherein potassium intercalates sequentially to form intermediate ternary selenides (CoKSe_2_, CoK_2_Se_2_, and CoK_3_Se_2_) before fully converting to metallic Co and K_2_Se Equations (4)–(7) [37]. The second route is the proposed conversion–insertion pathway based on phase diagram insights, where CoSe_2_ first converts to Co_3_Se_4_ and K_2_Se, followed by K^+^ insertion to form Co_3_KSe_4_, and finally decomposes into metallic Co along with potassium selenides K_2_Se and K_2_Se_3_ Equations (1)–(3).

(1) $$\mathrm{CoS}e_{2}+4/3K^{+}+4/3e^{-}\to 1/3Co_{3}Se_{4}+2/3K_{2}\mathrm{Se},E_{f}=-2.11\mathrm{eV}$$

(2)

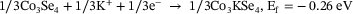



(3)

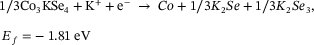



(4)

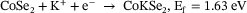



(5)

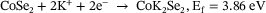



(6)

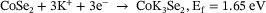



(7)

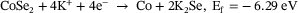




For each elementary step in both pathways, the formation energies (E_f_) were calculated to evaluate the thermodynamic favorability. The calculated E_f_ values for the proposed Co_3_Se_4_‐mediated route are −2.11, −0.26, and −1.81 eV for Equations (1)–(3), respectively, indicating consistent downhill energy profiles (Figure 1a). In contrast, the conventional K^+^ insertion pathway exhibits large positive formation energies of +1.63, +3.86, and +1.65 eV for the first three steps (Equations (4)–(6)), with only the final full conversion step, Equation (7), being energetically favorable (−6.29 eV) (Figure 1b). This comparison suggests that the Co_3_Se_4_‐mediated mechanism is thermodynamically more accessible and may serve as a lower‐strain reaction route during cycling.

**FIGURE 1:**
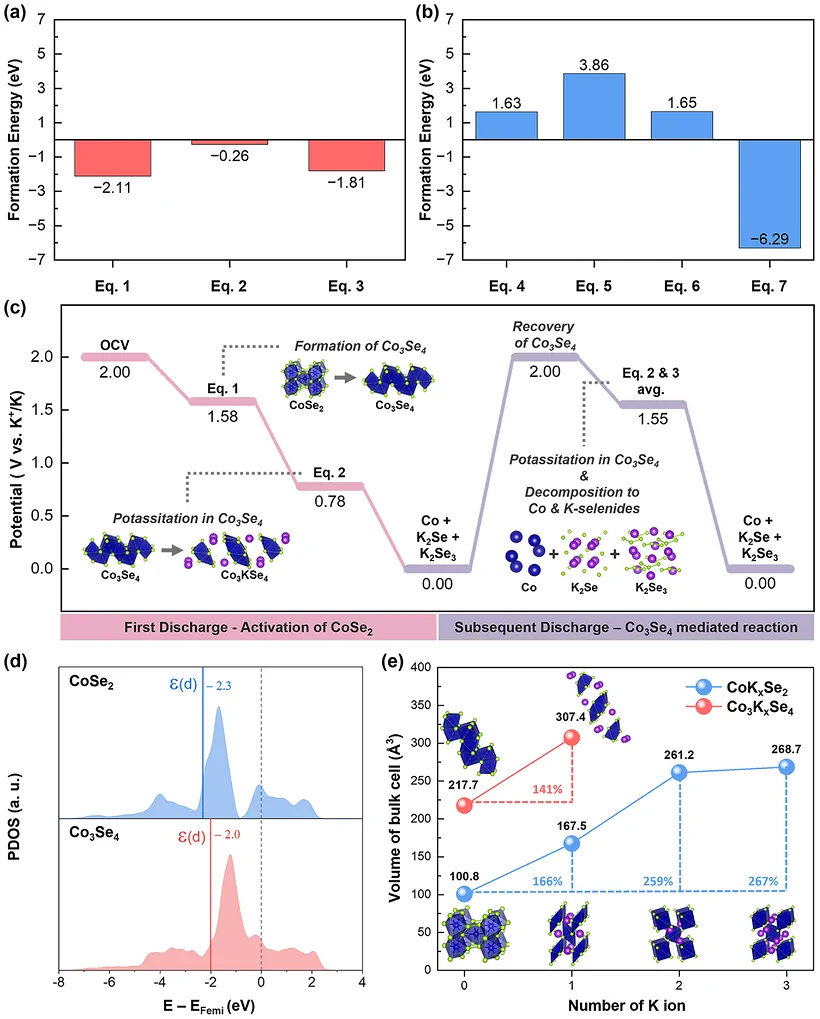
(a) Formation energies for Co_3_Se_4_‐mediated pathway (Equations (1)–(3). (b) Formation energies for conventional K^+^ insertion pathway into CoSe_2_ (Equations (4)–(7). (c) Theoretical voltage profiles and schematic reaction mechanisms for the first discharge (structural activation of CoSe_2_) and subsequent discharge (Co_3_Se_4_‐mediated reaction). (d) Projected density of states (PDOS) for Co atoms in CoSe_2_ and Co_3_Se_4_. (e) Relative volume expansion upon K^+^ insertion into CoSe_2_ and Co_3_Se_4_.

The thermodynamic feasibility of Co_3_Se_4_ formation, as identified through the phase diagram, aligns with the calculated formation energy, suggesting it is a highly favorable reaction pathway. However, formation energies at 0K alone have limitations in directly explaining phase transformation behaviors during electrochemical cycling at room temperature. Therefore, we converted the calculated E_f_ into theoretical potentials (V vs. K^+^/K) using the Nernst equation to conduct an in‐depth analysis of the feasibility of the proposed conversion reaction and the expected voltage profile (Figure 1c). First, the theoretical potential for the conversion of Co_3_Se_2_ to Co_3_Se_4_ according to Equation (1) was calculated to be approximately 1.58 V. The subsequent reactions, Equation (2) (representing the potassiation of Co_3_Se_4_ to the ternary intermediate Co_3_KSe_4_) and Equation (3) (corresponding to the further decomposition of Co_3_KSe_4_ into metallic Co and K‐selenides), exhibit theoretical potentials of 0.78 and 1.81 V, respectively. A noteworthy observation here is the voltage inversion phenomenon, where the potential for the decomposition of Co_3_KSe_4_ (1.81 V) is significantly higher than its formation potential (0.78 V). This indicates that Co_3_KSe_4_ is a thermodynamically metastable intermediate that, once formed, possesses a powerful driving force for immediate conversion into the final discharge products. In practice, the average potential of Equations (2) and (3) is approximately 1.55 V, predicting that the entire process following Co_3_Se_4_ formation proceeds rapidly along a steep downhill energy landscape, resulting in a single integrated voltage plateau around 1.55 V.

In addition to thermodynamic stability, electronic and structural factors further support the proposed Co_3_Se_4_‐mediated pathway. Projected density of states (PDOS) calculations revealed that the d‐band center of Co in Co_3_Se_4_ is located at −2.0 eV, closer to the Fermi level than in CoSe_2_ (−2.3 eV) (Figure 1d). This upward shift facilitates enhanced electronic hybridization between the K and Co_3_Se_4_ orbitals, effectively lowering the electronic barrier for potassiation. Bader charge analysis further quantifies this effect (Table S1). The K ion in the Co_3_Se_4_‐mediated intermediate transfers significantly more charge (+0.75 *e^−^*) than in the CoSe_2_‐mediated pathway (+0.49 *e^−^*). Notably, the Se atoms in Co_3_KSe_4_ achieve a highly anionic state (−0.52 *e^−^*), indicating a strong electrostatic interaction with K. Since this electronic state of Se is closely aligned with the chemical environment of the final potassium selenides, it provides a powerful thermodynamic driving force for the immediate decomposition of the metastable Co_3_KSe_4_ phase into metallic Co, K_2_Se, and K_2_Se_3_. This electronic transition offers a robust explanation for the voltage inversion phenomenon and the rapid reaction kinetics observed during the potassiation process.

To determine the stage at which the most critical volume expansion occurred, the calculated unit cell volumes for all intermediates in both reaction pathways were analyzed. During potassiation, volume changes associated with each intermediate phase significantly affect the structural stability of the electrode. The calculated unit cell volumes show that pristine CoSe_2_ has a volume of 100.8 Å^3^ (Figure 1e). Upon sequential K^+^ intercalation in the conventional insertion pathway, the volumes increased dramatically to 167.5 Å^3^ for CoKSe_2_ (166% increase compared with CoSe_2_), 261.2 Å^3^ for CoK_2_Se_2_ (259%), and 268.7 Å^3^ for CoK_3_Se_2_ (267%). These large volume expansions can induce severe mechanical strains and degrade the electrode structure. In contrast, the proposed conversion pathway through Co_3_Se_4_ exhibits markedly different volume‐change behaviors. Co_3_Se_4_ has a volume of 217.7 Å^3^, which already represents a more than two‐fold increase (approximately 216%) compared with pristine CoSe_2_. However, after the formation of Co_3_Se_4_, further K^+^ insertion to form Co_3_KSe_4_ resulted in volume expansion to 307.4 Å^3^, corresponding to an increase of approximately 141% compared with Co_3_KSe_4_ itself. This substantially smaller subsequent volume change indicates that the major mechanical strain was strongly localized at the initial CoSe_2_ → Co_3_KSe_4_ conversion step, while subsequent potassiation steps imposed considerably less structural stress. This disparity highlights the critical importance of controlling the large volume expansion at the initial conversion stage to preserve structural integrity and electrochemical performance. The pronounced initial volume expansion (∼216%) contrasts with the more moderate 141% expansion after Co_3_Se_4_ formation, suggesting that buffering this early transformation is key to improving cycling stability.

The DFT analysis clearly identified the initial CoSe_2_ → Co_3_Se_4_ conversion as the stage responsible for the most pronounced volume expansion and structural strain. To mitigate this critical bottleneck and maintain structural integrity during cycling, a strain‐tolerant electrode architecture was designed and synthesized. In this approach, HCS were interlinked with carbon nanotubes (CNTs) to form mechanically robust and highly conductive microclusters, which subsequently served as a host for CoSe_2_, yielding the SDHCS/CNT@CoSe_2_ composite. Figure 2a schematically illustrates the overall synthesis strategy for the SD‐SiO_2_@RF/CNT, SD‐HCS/CNT, and SD‐HCS/CNT@CoSe_2_ microclusters. Initially, SiO_2_@resorcinol–formaldehyde (RF) nanospheres (synthesized via an in situ Stöber templating method) were mixed with acid‐treated CNTs in an aqueous suspension at predetermined mass ratios (SiO_2_@RF: CNT = 2:1, 1:1, and 1:2), followed by spray‐drying to yield SD‐SiO_2_@RF/CNT microclusters [38]. As shown in the scanning electron microscopy (SEM) image in Figure S1, regardless of the mixing ratio, all resulting samples successfully formed spherical microclusters because the SiO_2_@RF nanospheres with a diameter of 200 nm were uniformly assembled rather than existing as individual particles. In all cases, the microclusters exhibit comparable sizes and morphologies, with their surfaces exhibiting a distinctive embossed texture attributed to the embedded SiO_2_@RF nanospheres. Additionally, CNTs were consistently interwoven throughout the microcluster surfaces, effectively binding the assembled nanospheres and enhancing mechanical cohesion. At lower CNT contents (for example, 2:1), individual CNT strands are clearly discernible, and the internal SiO_2_@RF nanospheres remain visible (Figure S1a,b). As the CNT ratio increased, the CNT network became denser and progressively obscured the nanospheres beneath it (Figure S1c,d). In the case of the 1:2 sample, CNTs formed a continuous sheath across the surface and completely masked the internal structure, resulting in a uniformly coated appearance (Figure S1e,f). These morphological observations suggest that insufficient CNT content may weaken the interparticle binding between nanospheres, whereas excessive CNT coverage could hinder ion diffusion by blocking surface pores. Therefore, tuning the CNT content is crucial for balancing structural integrity and electrochemical accessibility for optimal anode design. Based on the subsequent electrochemical evaluation of the PIB anode material, the sample with a SiO_2_@RF:CNT ratio of 1:2 achieved the most favorable performance. Consequently, this composition was selected for further structural and electrochemical investigation, as discussed in the following sections.

**FIGURE 2:**
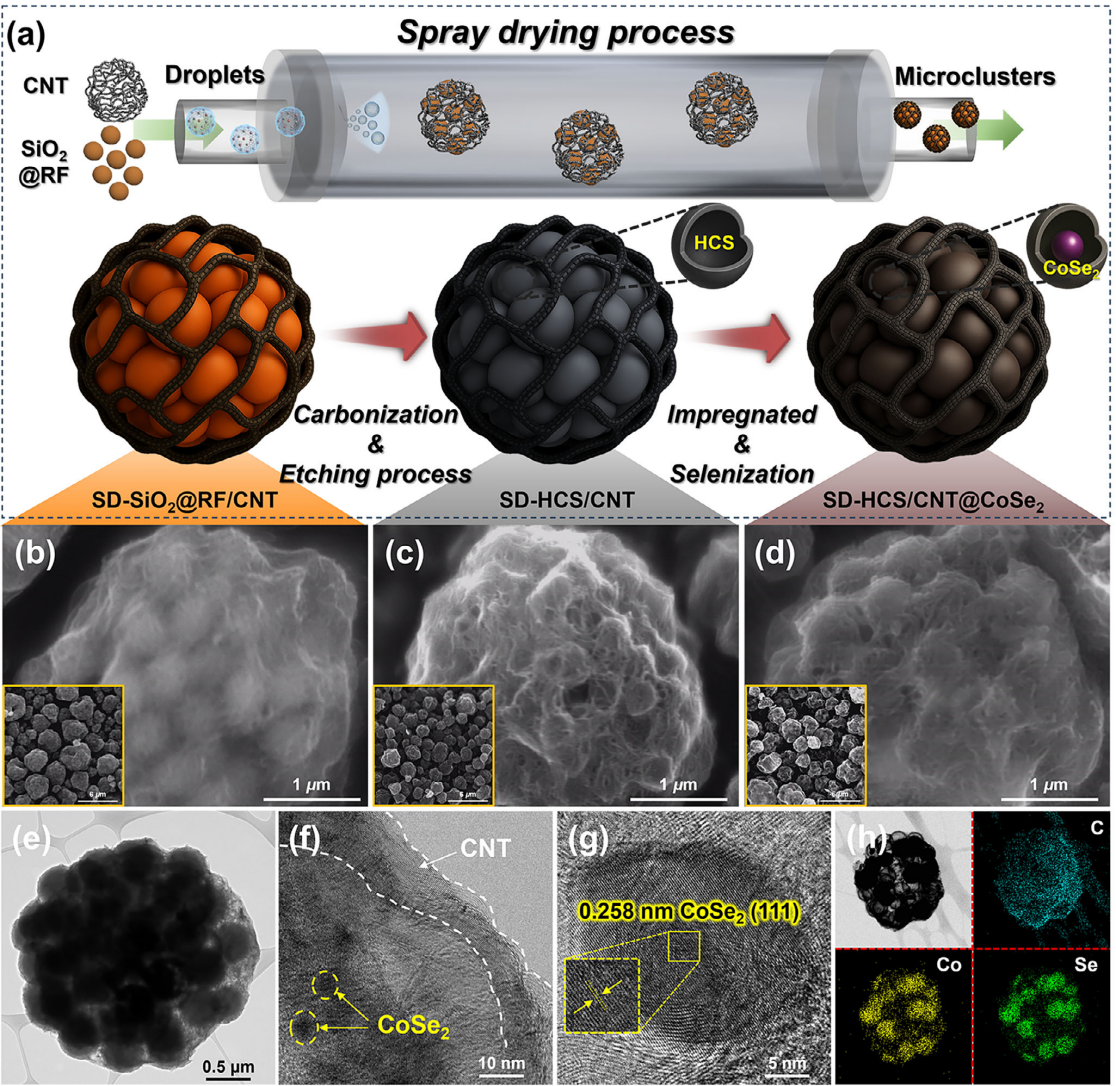
Schematic and morphological characterization of SD‐SiO_2_@RF/CNT, SD‐HCS/CNT, and SD‐HCS/CNT@CoSe_2_ microclusters. (a) Schematic illustration of the preparation process of SD‐HCS/CNT@CoSe_2_. Scanning electron microscopy (SEM) images of (b) SD‐SiO_2_@RF/CNT, (c) SD‐HCS/CNT, and (d) SD‐HCS/CNT@CoSe_2_ microclusters. (e, f) transmission electron microscopy (TEM) and (g) high‐resolution TEM (HR‐TEM) images of SD‐HCS/CNT@CoSe_2_ microclusters. (h) Energy dispersive spectroscopy (EDS) elemental maps of SD‐HCS/CNT@CoSe_2_.

Next, SD‐SiO_2_@resorcinol‐formaldehyde (RF) /CNT (Figure 2b) was thermally carbonized at 900°C under argon to convert RF into carbon, followed by chemical etching in an alkali solution to remove the sacrificial SiO_2_ core. The resulting SD‐HCS/CNT composite is shown in Figure 2c. After removing the SiO_2_ template, the HCS retained their spherical morphology and remained uniformly assembled into microclusters, with CNTs interwoven throughout the structure, forming a robust 3D conductive network (Figure 2c). The hollow nature of the HCS is confirmed by SEM images showing partially broken shells within the microclusters, exposing their empty interiors (Figure S2). This structural feature was further validated through transmission electron microscopy (TEM) analysis, which revealed a typical internal diameter of approximately 300 nm (Figure S3a). In the high‐magnification TEM images, intimate integration between the HCS and CNTs is clearly observed, with multiple HCS interconnected by CNTs throughout the cluster framework (Figure S3b). Furthermore, each HCS has a uniform shell thickness of approximately 30 nm (Figure S3c), within which well‐developed mesopores with diameters of approximately 10 nm are clearly visible (Figure S3d). For comparison, a control sample was prepared by mechanically mixing the HCS and CNT (M‐HCS/CNT) in a mortar and pestle. The SEM images of the physically blended M‐HCS/CNT mixture (Figure S4a) show randomly aggregated clusters, in contrast to the uniform spherical microclusters observed in SD‐HCS/CNT. At higher magnification (Figure S4b), individual HCS particles appear entangled in a web of CNTs without exhibiting consistent connectivity or ordered assembly. This contrasting morphology underscores the role of spray drying in producing well‐defined, CNT‐reinforced hollow spheres, providing a direct basis for evaluating the impact of microcluster architecture.

To assess the mechanical robustness of the synthesized SD‐HCS/CNT microclusters, ball milling tests were conducted for 15 min on the dextrin‐assembled SD‐HCS (without CNT) and CNT‐reinforced SD‐HCS/CNT samples (Figure S5). As shown in the postmilling SEM images, the SD‐HCS sample (Figure S5a,b) exhibited significant structural collapse and fragmentation under mechanical stress. In contrast, the CNT‐reinforced SD‐HCS/CNT microclusters (Figure S5c,d) retained their spherical morphology without noticeable damage, clearly demonstrating that the presence of interwoven CNTs effectively enhanced their structural integrity and resistance to mechanical impact.

To further assess the impact of CNT integration on the pore structure, nitrogen adsorption–desorption measurements were carried out on pristine HCS (not spray‐dried) and the SDHCS/CNT microclusters. As shown in Figure S6a,b, both samples exhibited the Type IV(b) isotherm characteristics of mesoporous materials; their textural parameters are listed in Table S1. Pristine HCS exhibits a BET surface area of 509.5 m^2^ g^−1^, total pore volume of 1.34 cm^3^ g^−1^, and volume‐weighted average pore diameter of 17.65 nm, with the latter value being skewed upward by a tail of very large (>150 nm) pores. After CNT incorporation, SDHCS/CNT exhibited an even higher surface area (608 m^2^ g^−1^) and pore volume (2.93 cm^3^ g^−1^), with an average pore diameter of 17.25 nm. The increase in area and volume is attributed to the formation of many additional mid‐sized pores (10–50 nm), because the interwoven CNT network subdivided or blocked the few extreme macropores in the HCS. This reorganization produced a pore hierarchy dominated by mesopores and small macropores that maximized the accessible surface area while slightly lowering the volume‐weighted average pore size. Despite the CNT binding, these enhanced textural properties facilitated the uniform infiltration of metal ion precursors into the hollow interiors. Moreover, the CNTs penetrated both the exterior and interior of each HCS, establishing a continuous 3D conductive network throughout the microclusters (Figure S3b).

To determine how the HCS: CNT ratio influences both the morphology and pore structure of the SD‐HCS/CNT hosts designed in this study, three pristine microcluster samples with HCS: CNT mass ratios of 2:1, 1:1, and 1:2 were synthesized. Subsequently, SEM imaging was performed alongside nitrogen adsorption–desorption measurements (Figure 3a–f). The SEM images revealed that, at a 2:1 ratio (Figure 3a), the interwoven CNT network was sufficiently sparse to allow the underlying HCS to be clearly visible and individually delineated. As the CNT content increased to 1:1 (Figure 3b), the spheres became progressively enveloped by CNTs, producing a denser sheath, but still allowing some pore openings to remain exposed. In the 1:2 sample (Figure 3c), the CNTs formed a near‐continuous coating that almost completely masked the HCS surface, suggesting the potential blockage of surface pores. These morphological trends are reflected in the Brunauer–Emmett–Teller (BET) data (insets of each panel, Figure 3d–f). The 2:1 sample exhibits the highest surface area (608 m2 g^−1^) and pore volume (2.93 cm3 g^−1^) with an average pore diameter of 17.25 nm, indicating that minimal CNT coverage preserved the intrinsic mesoporosity of the HCS while adding mid‐sized CNT‐derived voids (Figure 3d and Table S2). At a ratio of 1:1, the intermediate values of 358 m^2^ g^−1^, 1.91 cm^3^ g^−1^, and 19.58 nm reflect a balance between the preserved mesopores and partial CNT blockage (Figure 3e and Table S2). In contrast, the 1:2 sample exhibits the lowest surface area (251 m2 g^−1^) and pore volume (1.36 cm^3^ g^−1^) but the largest average pore diameter (20.14 nm), consistent with extensive CNT coverage that occludes smaller mesopores and leaves only the largest voids accessible (Figure 3f and Table S2). These observations indicate that excessive CNTs (1:2) formed a dense sheath, which blocked the mesopores and limited the accessible surface area, whereas an intermediate CNT loading (1:1) still obscured significant portions of the HCS pore network, potentially hindering uniform CoSe_2_ infiltration. In contrast, the lower CNT content in the 2:1 host preserved the mesoporous HCS structure, delivered the highest BET surface area and pore volume, and maintained sufficient CNT reinforcement. Therefore, the 2:1 sample was considered as the most suitable scaffold for the subsequent CoSe_2_ loading.

**FIGURE 3:**
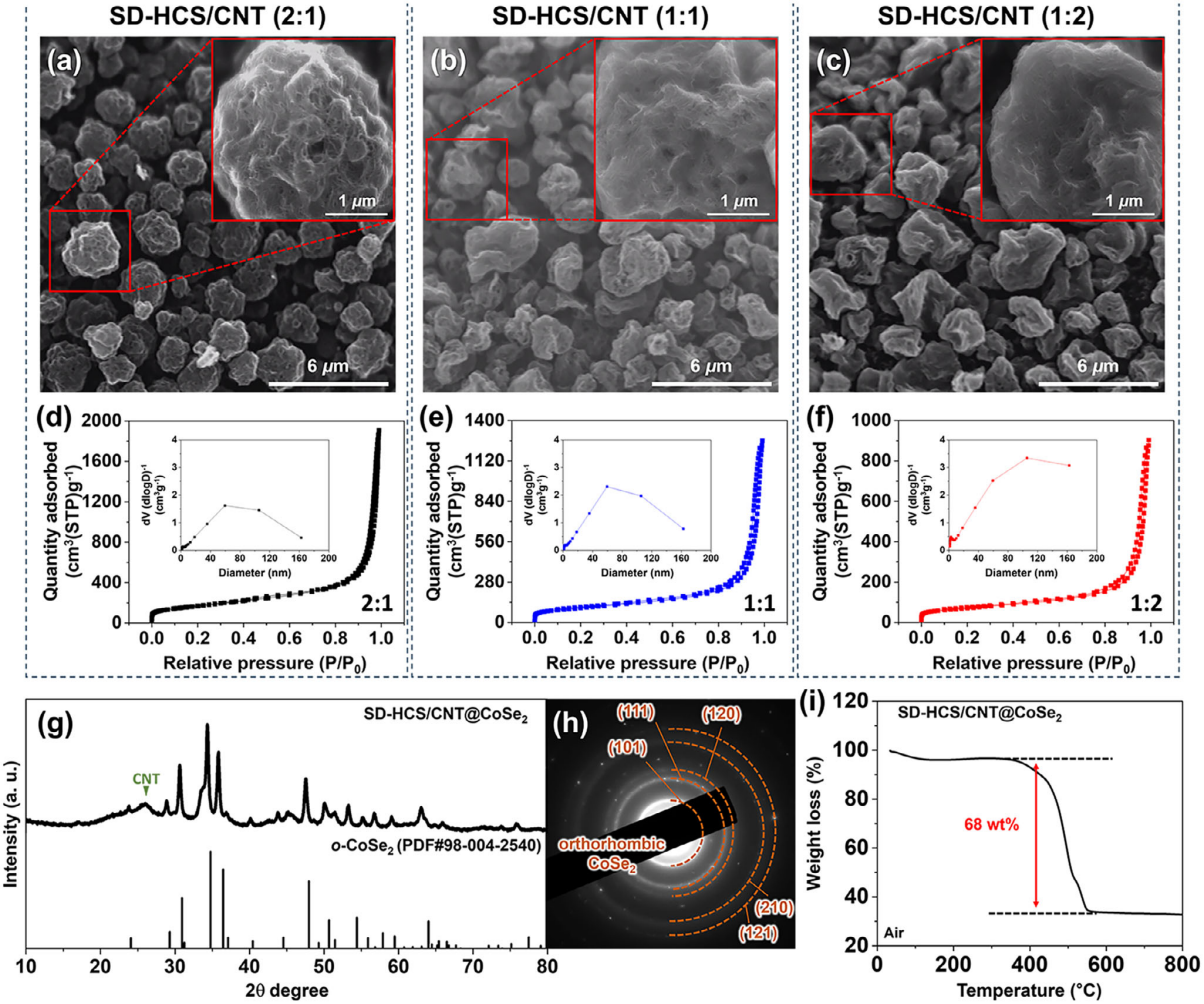
SEM images of SD‐HCS/CNT hosts at different HCS: CNT ratios: (a) 2:1, (b) 1:1, and (c) 1:2. N_2_ adsorption–desorption isotherms and textural properties of SD‐HCS/CNT hosts at different HCS: CNT ratios: (d) Type IV(b) isotherm for HCS: CNT = 2:1; (e) Type IV(b) isotherm for HCS: CNT = 1:1; (f) Type IV(b) isotherm for HCS: CNT = 1:2. Structural analysis of SD‐HCS/CNT@CoSe_2_. (g) XRD pattern of SD‐HCS/CNT@CoSe_2_ (PDF#98‐004‐2540); (h) Selected area diffraction (SAED) pattern of SD‐HCS/CNT@CoSe_2_. (i) thermogravimetric analysis of SD‐HCS/CNT@CoSe_2_ in air up to 800°C.

Building on this robust CNT‐woven host, SD‐HCS/CNT was impregnated with a Co precursor via wet infiltration, followed by selenization in an Ar/H2 atmosphere to yield SD‐HCS/CNT@CoSe_2_. After the infiltration of the cobalt precursor and selenization, the original spherical microclusters were well preserved (Figure 2d). Notably, distinct CoSe_2_ particles were not observed on the external surface, suggesting that the cobalt precursor effectively infiltrated the interior of the SD‐HCS/CNT microclusters and that selenization occurred uniformly within the hollow domains. In contrast, when the HCS:CNT ratio was reduced to 1:1 or 1:2, the CoSe_2_ nanoparticles failed to penetrate the hollow cores and were instead deposited on the outer surface of the microclusters (Figure S7a,b). Such surface‐exposed CoSe_2_ cannot be limited to large conversion‐induced volume changes, leading to rapid electrode degradation. In fact, cycle‐life tests (Figure S7c) revealed that the 1:1 and 1:2 samples suffered dramatic capacity losses after just 30 cycles, directly demonstrating the detrimental effect of the uncontained CoSe_2_ expansion on long‐term stability. These observations, along with BET and morphological analyses, confirm that the HCS: CNT ratio of 2:1 is the most suitable scaffold for effective CoSe_2_ loading.

In Figure 2e, the TEM image of SD‐HCS/CNT@CoSe_2_ shows that the hollow interiors of HCS, which appear empty in SD‐HCS/CNT (Figure S3a), are filled with CoSe_2_ nanoparticles. The spatial confinement of CoSe_2_ indicates that the internal cavities of the HCS serve as a robust template for hosting the active material, thereby mitigating the volumetric expansion typically associated with CoSe_2_ during cycling and maintaining the structural integrity of the microclusters. Moreover, this design promotes continuous electron transport through the interwoven CNT network, thereby enhancing the composite's electrical conductivity. In the high‐magnification TEM image (Figure 2f), CoSe_2_ nanoparticles with diameters of 4–10 nm are shown to be uniformly distributed throughout the HCS interiors. High‐resolution TEM (HR‐TEM) (Figure 2g) reveals distinct lattice fringes with an interplanar spacing of 0.258 nm, corresponding to the (111) plane of orthorhombic CoSe_2_. Additionally, the elemental mapping results in Figure 2h indicate overlapping Co and Se signals, further confirming the homogeneous distribution of CoSe_2_ throughout the internal structure.

Figure 3g presents the XRD pattern of SD‐HCS/CNT@CoSe2, wherein all diffraction peaks match exactly with the reference pattern for orthorhombic CoSe_2_ (*o*‐CoSe_2_) (PDF#98‐004‐2540, Hastite), except for the presence of CNTs, confirming its phase purity. Figure 3h shows the corresponding SAED pattern, where the observed rings correspond to *o*‐CoSe_2_, further verifying the crystalline nature of the loaded CoSe_2_ within the hollow carbon–CNT host.

To quantify the CoSe_2_ loading and evaluate the thermal stability of the synthesized SD‐HCS/CNT@CoSe_2_, thermogravimetric analysis was performed in air up to 800°C (Figure 3i). The sample underwent a total weight loss of approximately 68%, attributed to the combustion of carbon into CO_2_ and the conversion of cobalt species into Co_3_O_4_. From the Co_3_O_4_ residue, the CoSe_2_ content was determined to be 58.3 wt.%, which is in close agreement with the initially targeted 60 wt.%, confirming the successful infiltration and conversion of the Co precursor within the microcluster host.

Figure S8a shows the full survey spectrum of SD‐HCS/CNT@CoSe_2_, confirming the presence of C, cobalt, and selenium. In the high‐resolution C 1s spectrum (Figure S8b), three distinct components are observed at 284.6, 285.3, and 286.4 eV, corresponding to the C─C, C─O, and C═O bonds, respectively. The Co 2p spectra (Figure S8c) were deconvoluted into four main peaks and two shake‐up satellites: the Co 2p_3/2_ peak at 783.1 eV and the Co 2p_1/2_ peak at 797.0 eV correspond to the Se‐Co‐Se and Co─Co bonding in CoSe_2_. The weaker peaks at 779.9 and 796.8 eV are attributed to the Co─O species generated by partial surface oxidation to Co_3_O_4_. The satellites at ∼786.5 and ∼802.6 eV further confirm the Co oxidation state. In the Se 3d spectrum (Figure S8d), the doublet at 54.9 eV (Se 3d_5/2_) and 55.5 eV (Se 3d_3/2_) matches the characteristic binding energies of Se in CoSe_2_, confirming the presence of the CoSe_2_ phase.

To experimentally validate the proposed reaction pathway under electrochemical conditions, in situ Xray diffraction measurements were performed during the potassiation/depotassiation process using the as‐synthesized SD‐HCS/CNT@CoSe_2_. This real‐time structural analysis enabled the direct observation of phase evolution, allowing confirmation of the presence of Co_3_Se_4_ as an intermediate and the correlation of the measured lattice variations with theoretical predictions. Figure 4a presents a contour map of the in situ XRD patterns collected during the first discharge–charge cycle of SD‐HCS/CNT@CoSe_2_ in the range of 0.01–3.0 V. The intensity is indicated by a color gradient (warm tones for high intensity, cool tones for low intensity), and phase annotations mark the appearance of different cobalt selenide species. Before discharge (rest state), strong reflections at 2θ = 30.4°, 34.7°, and 35.7° correspond to the (110), (111), and (012) planes of *o*‐CoSe_2_.

**FIGURE 4:**
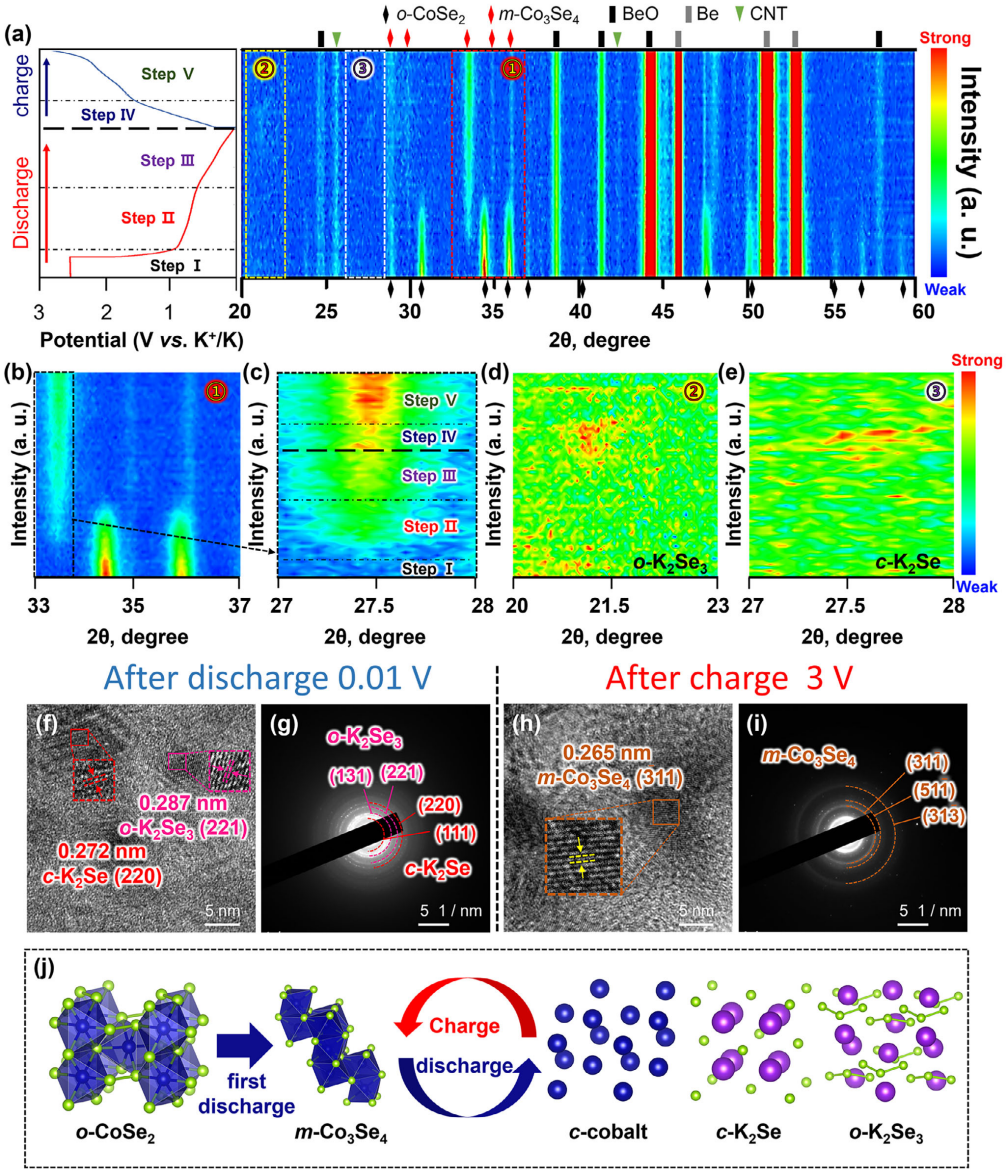
In situ XRD during first K^+^ discharge–charge cycle. (a) Contour plot of in situ XRD patterns collected in the range of 0.01–3.0 V, with color scale indicating diffraction intensity (warm = strong, cool = weak). (b, c) Expanded views of *o*‐CoSe_2_ → *m*‐Co_3_Se_4_ transition region at 33.7°. (d, e) Expanded views of faint *o*‐K_2_Se_3_ (region 2) and *c*‐K_2_Se (region 3). (f, g) TEM and SAED of microclusters after full discharge to 0.01 V. (h, i) TEM and SAED of microclusters after full charge to 3.0 V. (j) Schematic of reversible conversion pathway: *o*‐CoSe_2_ → *m*‐Co_3_Se_4_ → metallic Co + *o*‐K_2_Se_3_/*c*‐K_2_Se during discharge, and reconversion to *m*‐Co_3_Se_4_ upon charge.

As discharge proceeded (Step II), these *o*‐CoSe_2_ peaks gradually diminished, and a new reflection emerged at 33.7° (Figure 4b), indicating the formation of monoclinic Co_3_Se_4_ (*m*‐Co_3_Se_4_) as an intermediate. Interestingly, while the peak of *m*‐Co_3_Se_4_ was observed starting from approximately 1.48 V (close to the theoretical potential of 1.58 V), the subsequent coexistence of both phases occurred primarily between 1.0 and 0.5 V (Step II), with the complete disappearance of the *o*‐CoSe_2_ peaks occurring at 0.5 V. This discrepancy indicates a substantial kinetic overpotential (> 0.5 V) associated with the structural reorganization of the bulk *o*‐CoSe_2_ into the *m*‐Co_3_Se_4_ phase. The magnification of the *m*‐Co_3_Se_4_ reflection (Figure 4c) reveals that its intensity remained low during discharge (Steps II–III). This phenomenon can be attributed to the rapid consumption of the *m*‐Co_3_Se_4_ phase upon its formation, driven by the kinetics of the subsequent reaction steps. Specifically, the higher theoretical potential for the final decomposition (Equation (3), 1.81 V) compared to that for Co_3_KSe_4_ formation (Equation (2), 0.78 V) creates a strong thermodynamic driving force that triggers a cascade‐like reaction toward the final discharge products once the initial potassiation of *m*‐Co_3_Se_4_ begins. Consequently, *m*‐Co_3_Se_4_ reacts immediately with K^+^ to reach the final discharge state without significant accumulation, maintaining a low steady‐state concentration throughout the initial conversion process. In contrast, the prominent intensification of the *m*‐Co_3_Se_4_ reflection during the subsequent charging process (Steps IV–V) demonstrates the high reversibility of the conversion reaction. The persistent recurrence of the *m*‐Co_3_Se_4_ phase in subsequent cycles further validates its role as the primary reversible active species within the electrochemical system (Figures S9 and S10).

A notable feature in the electrochemical profiles is the distinct voltage shift observed after the initial cycle. During the first discharge, the voltage plateau appears to the range of 1.0–0.5 V, however, from the second cycle onward, the discharge plateau shifts upward to the 1.4–1.2 V range (Figures S10b). This shifted plateau shows remarkable agreement with the calculated average potential of 1.55 V for the *m*‐Co_3_Se_4_‐mediated reaction pathway (the mean value of Equations (2) and (3), Figures S10a). Such a correlation suggests that the initial cycle serves as a structural activation process, after which the electrochemical energy storage is predominantly governed by the reversible transition of the *m*‐Co_3_Se_4_ phase. Therefore, the close match between the experimental operating voltage and theoretically calculated potential provides definitive evidence that *m*‐Co_3_Se_4_ is the key active species delivering the reversible capacity. Finally, the faint features observed in regions 2 and 3 in Figure 4a (expanded in Figure 4d,e) correspond to the orthorhombic K_2_Se_3_ (*o*‐K_2_Se_3_) and cubic K_2_Se (*c*‐K_2_Se_3_) discharge products, respectively, which formed after the *o*‐CoSe_2_ conversion reaction was complete. These in situ XRD results not only validate the DFT prediction of Co_3_Se_4_ as the key intermediate phase but also demonstrate that the electrochemical conversion initiated with CoSe_2_ and subsequently proceeded reversibly through the Co_3_Se_4_ phase. The mechanical constraint of the robust SD‐HCS/CNT host may exert localized compressive stress during potassiation, which, together with the large ionic radius of K^+^, kinetically retards the complete phase transformation of o‐K_2_Se_3_ into c‐K_2_Se. The unique advantage of this CNT‐interwoven architecture lies in its ability to promote electrochemical homogeneity. The long‐range electronic connectivity provided by the CNT network ensures a uniform potential distribution across the entire microcluster. This synchronicity is a prerequisite for the regulated Co_3_Se_4_‐mediated conversion. Unlike other carbon scaffolds that may cause localized resistance variations and obscure metastable phases, the CNT network effectively stabilizes the hidden reaction pathway by maintaining quasi‐equilibrium conditions throughout the electrode. These in situ XRD results not only validate the DFT prediction of Co_3_Se_4_ as the key intermediate phase but also demonstrate that the electrochemical conversion initiated with CoSe_2_ and subsequently proceeded reversibly through the Co_3_Se_4_ phase.

Complementing the in situ XRD results, the SDHCS/CNT@CoSe_2_ microclusters harvested at 0.01 V (fully discharged) and 3.0 V (fully charged) were examined using TEM and SAED to further confirm the identity and distribution of conversion products. Even after complete discharge to 0.01 V and full charge to 3.0 V, the low‐magnification TEM images (Figure S11a–d) confirm that the spherical microcluster architecture remained largely intact after both discharge and charge cycles, although the phase transition induced by the conversion reactions cannot be discerned. The HR‐TEM image (Figure 4f) shows lattice spacings of ∼0.287 and ∼0.272 nm, corresponding to the (221) plane of *o*‐K_2_Se_3_ and the (220) plane of *c*‐K_2_Se, respectively. The SAED pattern in Figure 4g further confirms these assignments, and the emergence of multiple diffraction rings indexed to *o*‐K_2_Se_3_ and *c*‐K_2_Se demonstrates that *o*‐CoSe_2_ underwent conversion to metallic Co and potassium selenide intermediates during discharge. It is noteworthy that while the diffraction peaks of o‐K_2_Se_3_ and c‐K_2_Se were clearly identified at the end of discharge, explicit peaks for metallic Co were not observed. This is attributed to the formation of ultra‐fine Co nanocrystals or a pseudo‐amorphous metallic state highly dispersed within the K_2_Se_x_ matrix and the carbon framework, a phenomenon frequently reported in conversion‐type metal selenide anodes [39]. After recharging to 3.0 V, HRTEM (Figure 4h) revealed a lattice fringe of ∼0.265 nm, corresponding to the (311) plane of *m*‐Co_3_Se_4_. The SAED pattern (Figure 4i) exhibits fewer rings compared with those observed after discharge, with the observed rings indexed to the *m*‐Co_3_Se_4_ phases. These results confirm that, upon recharging, the K‐selenide phases underwent a conversion reaction and phase transition to form a *m*‐Co_3_Se_4_ intermediate, rather than directly reverting to *o*‐CoSe_2_. Combined with the in situ XRD results, the ex situ TEM and SAED analyses revealed a consistent reversible conversion pathway: *o*‐CoSe_2_ → metallic Co + *o*‐K_2_Se_3_/*c*‐K_2_Se during discharge, followed by recombination to *m*‐Co_3_Se_4_ during the charge process. Although the formation of metallic Co is strongly supported by the TEM/SAED results and the proposed reaction pathway, its direct observation in operando XRD remains challenging. Furthermore, it is important to consider that the detection of metallic Co in operando XRD measurements can be inherently limited by instrumental factors. In particular, diffraction peaks of Co may overlap with signals from the BeO window used in the operando cell configuration, which can obscure weak Co reflections even when present [23]. The schematic of the entire conversion sequence is shown in Figure 4j, illustrating the stepwise transformation of *o*‐CoSe_2_ into *m*‐Co_3_Se_4_ upon the first discharge, after which *m*‐Co_3_Se_4_ and *c*‐K_2_Se/o‐K_2_Se_3_ underwent reversible conversion during subsequent cycling. The distinct Co_3_Se_4_ signals in our operando XRD are fundamentally enabled by the SD‐HCS/CNT architecture. This CNT‐interwoven mechanical cage effectively buffers the severe lattice strain during K‐ion insertion, preventing the reaction from bypassing the intermediate state. Additionally, the optimized network ensures low overpotential and reaction synchronicity across the microsphere, providing a quasi‐equilibrium environment for the regulated step‐wise transformation. Thus, the CNT framework acts as a kinetic regulator that stabilizes the hidden reaction pathway, rather than merely serving as a conductive path.

To elucidate the potassium‐ion storage behavior and charge‐transport kinetics of SD‐HCS/CNT@CoSe_2_, comprehensive electrochemical measurements were performed, employing cyclic voltammetry (CV), electrochemical impedance spectroscopy (EIS), the galvanostatic intermittent titration technique (GITT), and capacitive contribution analysis (Figure 5). First, the CV test was used to probe the potassium‐ion storage characteristics of SD‐HCS/CNT@CoSe_2_, with measurements conducted in the range of 0.01–3.0 V at a scan rate of 0.1 mV s^−1^ (Figure 5a). During the first cathodic sweep, a pronounced reduction peak appeared at approximately 0.6 and 0.3 V, attributed to the intercalation and transformation processes of CoSe_2_, coupled with the formation of a solid‐electrolyte interphase (SEI) layer [40]. Two distinct oxidation peaks emerged in the subsequent anodic sweep, corresponding to the reconversion of potassium selenide back to the cobalt selenide phase [38, 41]. These features confirm the characteristic conversion reaction of CoSe_2_ in PIBs. By the second and fifth cycles, the reduction peak at ∼0.6 V diminished, while new peaks at approximately 0.4 and 1.3 V became prominent in the cathodic sweep. This shift suggests the establishment of a more stable SEI layer after the initial cycle and that the cobalt selenide conversion reaction proceeded in a slightly altered manner once the electrode surface was passivated [42]. Therefore, the CV curves collectively confirm the reversible conversion reaction of cobalt selenide and the evolving electrochemical processes accompanying the SEI formation in the SD‐HCS/CNT@CoSe_2_ electrode. Figure S12a shows the CV result for M‐HCS/CNT@CoSe_2_ at a scan rate of 0.1 mV s^−1^, revealing redox features similar to those observed in the SD‐HCS/CNT@CoSe_2_ microclusters. The CV profile of M‐HCS/CNT@CoSe_2_ exhibits essentially the same redox peaks as those of SD‐HCS/CNT@CoSe_2_, indicating an analogous cobalt selenide conversion mechanism. In contrast, SD‐HCS/CNT does not exhibit conversion peaks, and only a broad cathodic signal is observed at ≈1.0 V owing to SEI formation and the featureless curves characteristic of double‐layer charging (Figure S12b).

**FIGURE 5:**
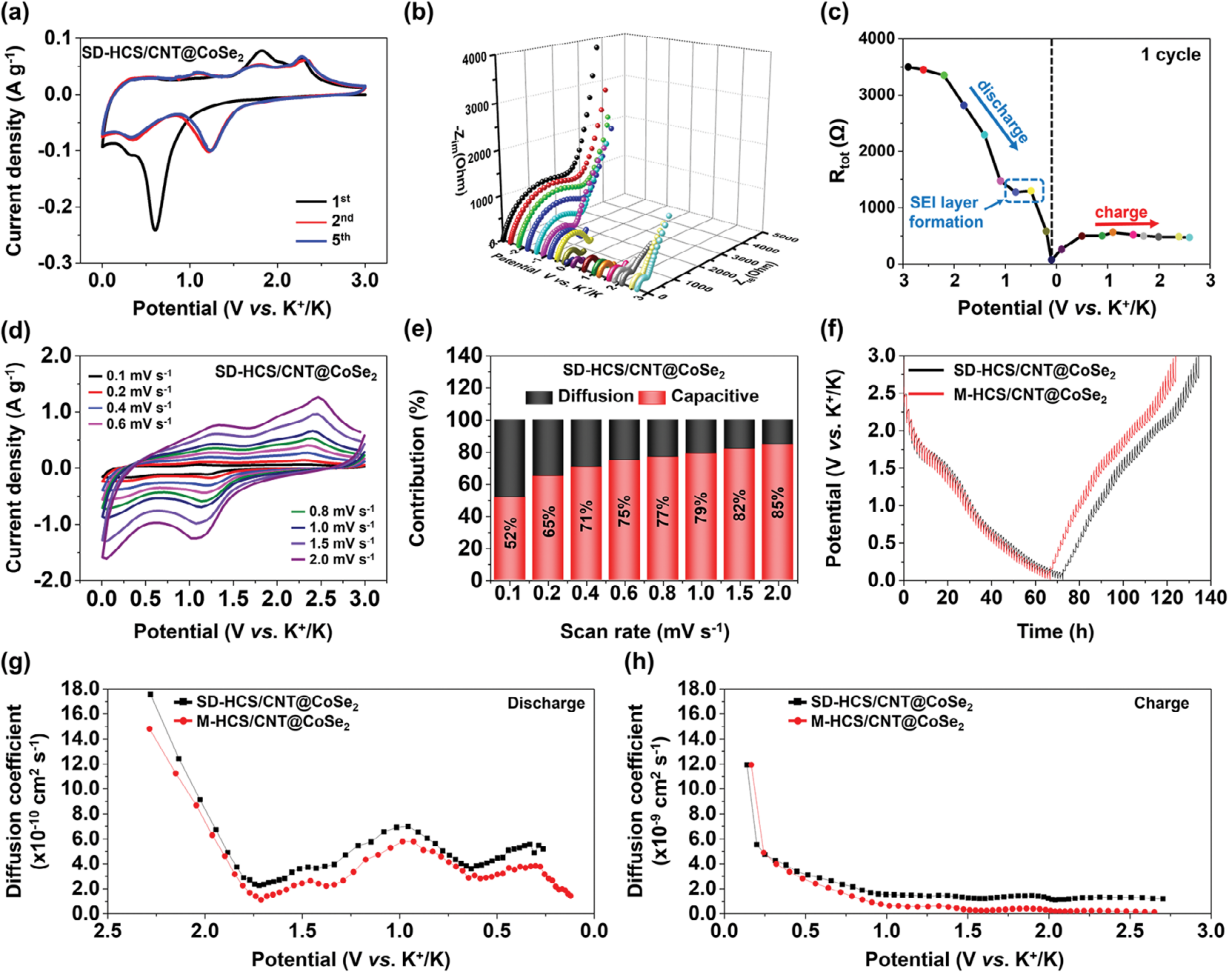
Electrochemical characterization of SD‐HCS/CNT@CoSe_2_. (a) Cyclic voltammetry (CV) curves at 0.1 mV s^−1^. (b) In situ electrochemical impedance spectroscopy (EIS) Nyquist plots recorded during the first discharge–charge cycle (0.01–3.0 V), with (c) extracted total resistance (R_tot_) profiles. (d) CV curves at varying scan rates (0.1–2.0 mV s^−1^) for capacitive contribution analysis. (e) Capacitive fraction versus scan rate for SD‐HCS/CNT@CoSe_2_ electrodes. (f) Galvanostatic intermittent titration technique (GITT) profiles and (g, h) K^+^ diffusion coefficients during 20th cycle for both electrodes.

To gain further insight into the electrochemical conversion of the SD‐HCS/CNT@CoSe_2_ electrode, in situ EIS was conducted throughout the first discharge–charge cycle (Figure 5b,c). The measurement was initiated at an open‐circuit potential of −2.5 V and conducted over the frequency range of 0.01–100 kHz, using a Randles‐type equivalent‐circuit model to extract the total resistance (R_tot_). The scan rate was set to 0.05 A g^−1^. In this model, R_tot_ is determined by summing the series resistance (R_s_), charge‐transfer resistance (R_ct_), and SEI resistance (R_sei_); the ion diffusion in the electrode also influences the overall impedance response. As shown in Figure 5c, R_tot_ continuously decreased during the discharge process, possibly owing to the conversion of CoSe_2_ into metallic Co, thereby improving electrical conductivity. However, between approximately 1.1 and 0.5 V, the decrease in R_tot_ is less pronounced. In this potential range, an SEI layer, which is often characterized by lower conductivity, formed concurrently with the CoSe_2_ → Co transformation. Consequently, the onset of SEI formation moderated the decrease in resistance, leading to a relatively flat region in the R_tot_ profile. During the subsequent charge process, R_tot_ increased again up to approximately 1.1 V, which can be explained by the reconversion of metallic Co back to CoSe_x_, a phase with lower conductivity compared with metallic Co, coupled with structural stress within the electrode. Above 1.1 V, R_tot_ began to decrease, presumably owing to volumetric contraction upon depotassiation and the partial decomposition or reorganization of the SEI layer.

Capacitive contribution analysis based on CV was carried out at various scan rates in the range of 0.1–2.0 mV s^−1^ to assess the reaction kinetics of both the SD‐HCS/CNT@CoSe_2_ and M‐HCS/CNT@CoSe_2_ electrodes (Figure 5d,e). As shown in Figure 5d, SD‐HCS/CNT@CoSe_2_ maintained well‐defined redox peaks even up to 2.0 mV s^−1^, demonstrating excellent high‐rate operability. By contrast, the M‐HCS/CNT@CoSe_2_ electrode began to lose its characteristic CV response at 1.0 mV s^−1^ and was essentially inactive at higher scan rates (Figure S12c). This decline in the high‐rate performance of M‐HCS/CNT@CoSe_2_ is attributed to its inability to effectively buffer the volume changes of CoSe_2_ during rapid K^+^ insertion/extraction, leading to degraded ion and electron transport pathways under fast‐scan conditions. To quantify the pseudocapacitive contribution, the current response was separated into diffusion‐controlled and capacitive components at each scan rate (Figure 5e and Figure S12d). At 0.8 mV s^−1^, SD‐HCS/CNT@CoSe_2_ exhibits a capacitive contribution of ∼77%, whereas M‐HCS/CNT@CoSe_2_ exhibits only ∼37%. Across all tested scan rates, SD‐HCS/CNT@CoSe_2_ consistently exhibits a higher capacitive fraction, confirming that its 3D CNT‐reinforced hollow carbon structure facilitated rapid charge storage through surface or near‐surface processes. Such a large pseudocapacitive contribution is advantageous for high‐rate performance, highlighting the ability of SD‐HCS/CNT@CoSe_2_ to sustain fast charge–discharge reactions through a capacitive‐controlled mechanism.

To gain additional insight into the potassium ion storage kinetics, the GITT was used to determine the diffusion coefficient of K^+^ (*D*_K_^+^) for SD‐HCS/CNT@CoSe_2_ and M‐HCS/CNT@CoSe_2_ (Figure 5f–h). The K^+^ diffusion coefficients for SD‐HCS/CNT@CoSe_2_ and M‐HCS/CNT@CoSe_2_ were evaluated after 20 cycles at 0.05 A g^−1^ using the GITT (Figure 3f), and *D*_K_^+^ was derived as follows:

$$D=\frac{4}{\pi \tau }{\left(\frac{V_{M}m_{B}}{M_{B}A}\right)}^{2}{\left(\frac{\Delta E_{s}}{\Delta E_{\tau }}\right)}^{2}$$

Here, *m*_B_, *V*_M_, and *M*_B_ denote the mass, molar volume, and molar mass of the active material, respectively; A is the electrode's geometric area; τ is the current pulse time (Figure S13). The changes in the steady‐state voltage (∆*E*_τ_) and overall voltage at each step (∆*E*_s_) can be calculated using experimental data and the properties of the electrode [43, 44]. Generally, *D*_K_^+^ decreased gradually throughout the charge–discharge process, except in specific voltage regions where a marked rise occurred. Notably, the higher *D*_K_^+^ in the mid‐to low‐voltage range during the discharge step is attributed to the phase transition of CoSe_2_ into Co, K_2_Se, and K_2_Se_3_, which temporarily enhanced ionic mobility [25, 45]. Across most potentials, the SD‐HCS/CNT@CoSe_2_ electrode exhibits higher *D*_K_^+^ values compared with M‐HCS/CNT@CoSe_2_, implying enhanced reaction kinetics in the spray‐dried microclusters (Figure 5g,h). This improvement is attributed to the robust 3D conductive network formed by the uniformly distributed CNT on the assembled HCS, which lowers the energy barrier for K^+^ transport. In contrast, the M‐HCS/CNT@CoSe_2_ electrode, which lacked the same level of structural uniformity, underwent comparatively limited ion diffusion, reflecting an overall less‐efficient pathway for K^+^ migration.

To investigate the K^+^ storage behavior and performance of the SD‐HCS/CNT@CoSe_2_ microclusters and other control groups, half‐cells were assembled using K metal as the counter electrode and potassium bis(fluorosulfonyl) imide (KFSI) salt in diethylene glycol dimethyl ether. Figure S14 compares the galvanostatic charge and discharge curves of SD‐HCS/CNT@CoSe_2_, M‐HCS/CNT@CoSe_2_, and SD‐HCS/CNT at a current density of 0.1 A g^−1^. The SD‐HCS/CNT@CoSe_2_ electrode exhibits a long, flat plateau centered around 0.75 V, reflecting a uniform K^+^ insertion into CoSe_2_ within the well‐encapsulated CNT‐bound HCS. In contrast, M‐HCS/CNT@CoSe_2_ exhibits a less pronounced plateau that begins at a higher voltage, indicating that some CoSe_2_ is deposited on the outer surface and directly exposed, and thus reacts earlier and less uniformly under the same conditions. In SDHCS/CNT@CoSe_2_, CoSe_2_ is homogeneously confined within the CNT‐wrapped HCS; therefore, its conversion reaction is initiated at a slightly lower potential, yielding a more uniform, lower‐voltage plateau. Moreover, the SD‐HCS/CNT electrode exhibits the characteristic sloping curve of a carbon‐only material, consistent with non‐Faradaic double‐layer charging and minor intercalation. The initial charge–discharge data reveal that SD‐HCS/CNT@CoSe_2_ delivered a discharge capacity of 537 mA h g^−1^ and charge capacity of 358 mA h g^−1^, corresponding to a CE of ∼66%. M‐HCS/CNT@CoSe_2_ provided a slightly lower initial discharge capacity of 504 mA h g^−1^ and charge capacity of 342 mA h g^−1^ (CE ∼67%). The higher capacity of SD‐HCS/CNT@CoSe_2_ is attributed to its superior K^+^ storage capability and enhanced ion diffusion, which result from the robust assembled HCS and effective dispersion of CoSe_2_. However, the marginally lower CE of SD‐HCS/CNT@CoSe_2_ likely stems from a greater degree of electrolyte decomposition and irreversible side reactions, which are exacerbated by the higher number of active sites available for ion insertion [46]. Figure 6a compares the cycling behaviors of SD‐HCS/CNT@CoSe_2_, M‐HCS/CNT@CoSe_2_, and SD‐HCS/CNT. The SD‐HCS/CNT@CoSe_2_ electrodes underwent five initial formation cycles at 0.1 A g^−1^, after which all subsequent performance data were collected at 0.5 A g^−1^. The SD‐HCS/CNT@CoSe_2_ microclusters exhibit a gradual capacity increase up to the 100th cycle, reaching ∼370 mA h g^−1^ and maintaining this value through the 200th cycle. Notably, the CE exceeded 99% during the formation cycles, indicating effective SEI formation and stable electrode operation. In contrast, M‐HCS/CNT@CoSe_2_ underwent an initial capacity increase up to ∼350 mA h g^−1^ at the 60th cycle, followed by capacity fading thereafter. The corresponding charge–discharge profiles (Figure 6b,c) reveal that SD‐HCS/CNT@CoSe_2_ exhibits more stable voltage curves compared with M‐HCS/CNT@CoSe_2_, whose 150^th^ cycle profile deviates significantly from earlier cycles. This underscores the importance of designing a robust electrode structure: the spray‐dried SD‐HCS/CNT configuration ensures that CoSe_2_‐impregnated HCS are uniformly bound by CNT, mitigating structural stress and buffering volumetric expansion during repeated K^+^ insertion and extraction. In contrast, M‐HCS/CNT@CoSe_2_ lacks these spray‐dried agglomerates and is characterized by randomly clustered HCS and CNT domains (Figure S4). This results in uneven stress and CoSe_2_ distributions, leading to electrode degradation and eventually to poor cycling stability. The carbon‐only SD‐HCS/CNT electrode exhibits a lower capacity (typical of carbon‐based materials), reflecting the absence of a CoSe_2_ conversion reaction. The initial increase in capacity, often termed the activation process, is primarily attributed to the gradual electrolyte infiltration into the hierarchical SD‐HCS/CNT clusters. This enables the hidden active sites inside the hollow carbon host to participate in the K^+^ storage reaction. Additionally, the in situ structural refinement into smaller nanocrystals during the first few cycles increases the effective surface area for electrochemical reactions [47].

**FIGURE 6:**
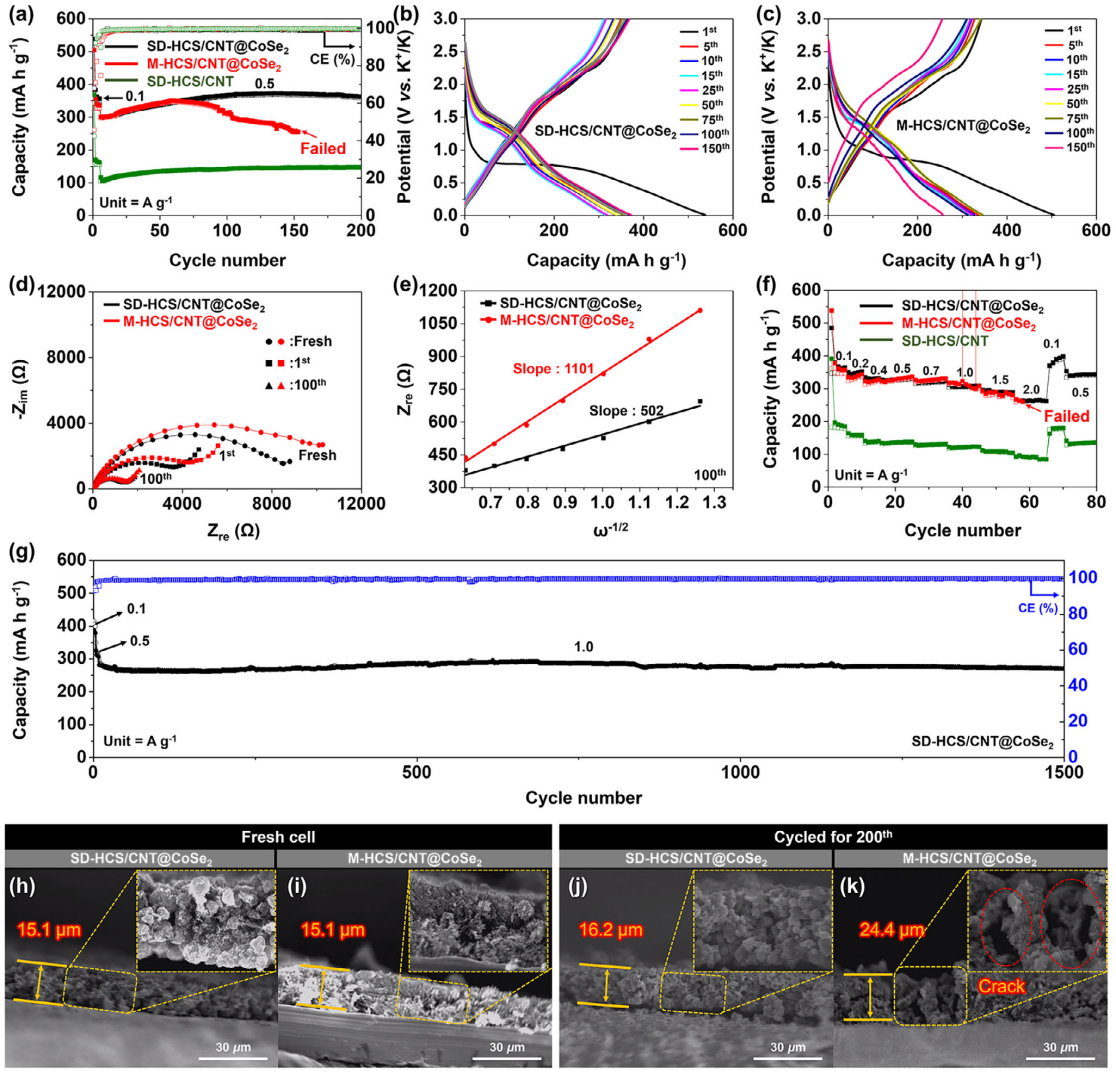
Half‐cell electrochemical setup and initial performance of SD‐HCS/CNT@CoSe_2_, M‐HCS/CNT@CoSe_2,_ and SD‐HCS/CNT. (a) Cycling performance at 0.5 A g^−1^, (b, c) Galvanostatic charge‐discharge (GCD) profiles at selected cycles of SD‐HCS/CNT@CoSe_2_ and M‐HCS/CNT@CoSe_2_, (d) Nyquist plot from selected cycles, (e) EIS angular‐frequency calculation data (Warburg impedance), (f) Rate capability at various current densities, (g) Long‐term cycling performance of SD‐HCS/CNT@CoSe_2_ at a high current density of 1.0 A g^−1^, (h–k) Cross‐sectional SEM images of SD‐HCS/CNT@CoSe_2_ and M‐HCS/CNT@CoSe_2_ electrodes before and after 200 cycles.

Building on the cycling results, the way in which the charge‐transfer resistance evolved with cycling was investigated by conducting ex situ EIS on fresh electrodes after one cycle and after 100 cycles (Figure 6d). Even in the fresh state, before SEI formation, SD‐HCS/CNT@CoSe_2_ exhibits a lower R_ct_ compared with M‐HCS/CNT@CoSe_2_. This behavior reflects the uniform spray‐drying‐induced CNT network that tightly binds to the HCS, creating a continuous 3D conductive scaffold. In contrast, in M‐HCS/CNT@CoSe_2_, the random mechanical mixing of HCS and CNT led to uneven contacts and poorer overall conductivity, hindering ion transfer and increasing R_ct_. After the first cycle, which included SEI formation, SD‐HCS/CNT@CoSe_2_ again exhibited a consistently lower R_ct_ compared with M‐HCS/CNT@CoSe_2_, and this trend persisted even after 100 cycles. In addition to charge transfer resistance, the K^+^ diffusion kinetics were evaluated by considering the Warburg region of the ex situ EIS data (Figure 6e). The diffusion slope for SDHCS/CNT@CoSe_2_ is 502 Ω s^−1/2^, which is less than half that of MHCS/CNT@CoSe_2_ (1101 Ω s^−1/2^), indicating significantly faster ion transport within the spray‐dried, CNT‐reinforced hollow framework. Combined with the lower R_ct_ values in the fresh, first‐cycle, and 100th cycle states, these results confirm that the SDHCS/CNT architecture markedly enhances both the charge transfer and diffusion kinetics during extended cycling.

The rate performance of the SD‐HCS/CNT@CoSe_2_ electrode was systematically evaluated at stepwise current densities of 0.1, 0.2, 0.4, 0.5, 0.7, 1.0, 1.5, and 2.0 A g^−1^ (Figure 6f). When subjected to current densities in the range of 0.1–2.0 A g^−1^, SD‐HCS/CNT@CoSe_2_ delivered capacities of ∼363, 347, 330, 329, 320, 306, 290, and 264 mA h g^−1^, respectively, subsequently recovering to 398 mA h g^−1^ (0.1 A g^−1^) and 350 mA h g^−1^ (0.5 A g^−1^). In contrast, M‐HCS/CNT@CoSe_2_ could not sustain operation at 2.0 A g^−1^, failing to cycle under such high current. This dramatic drop is not caused by an intrinsic limitation of CoSe_2_, but rather from the absence of a uniform, spray‐dried CNT host: in M‐HCS/CNT@CoSe_2_, HCS and CNT are simply mixed, leaving many CoSe_2_‐rich regions mechanically unsupported and unable to buffer the severe volumetric changes during rapid K^+^ insertion/extraction. Without the continuous 3D conductive network and stress‐relieving voids provided by the well‐bound CNT–HCS architecture in SD‐HCS/CNT@CoSe_2_, local electrode collapse and loss of electrical connectivity occurred at high rates, leading to failure. By comparison, SD‐HCS/CNT exhibits lower capacity, such as 187 mA h g^−1^ at 0.1 A g^−1^, consistent with the typical response of carbon‐based electrodes lacking a conversion‐type active material. Collectively, these results confirm the critical role of spray drying in producing a CNT‐bound hollow carbon host that ensures uniform CoSe_2_ distribution and withstands high‐rate cycling without significant performance degradation. This superior electrochemical performance is further highlighted by a comprehensive comparison with previously reported data, as summarized in Table S3.

To further evaluate the long‐term durability of the m‐Co_3_Se_4_ mediated pathway, extended cycling was performed at a high current density of 1.0 A g^−1^ (Figure 6g). The SD‐HCS/CNT@CoSe_2_ electrode demonstrated outstanding structural robustness, maintaining a stable reversible capacity of 270 mA h g^−1^ even after 1500 cycles. This exceptional longevity is attributed to the hierarchical CNT‐interwoven microcluster architecture, which effectively buffers the initial structural strain and prevents electrode pulverization during prolonged cycling. These results confirm that the stabilization of the Co_3_Se_4_ intermediate, facilitated by rational host design, is a key strategy for ensuring the structural integrity and reaction reversibility of conversion‐type anodes over a large number of cycles.

Next, the structural design of SD‐HCS/CNT@CoSe_2_ was investigated in detail to elucidate how it suppresses electrode swelling during cycling. Cross‐sectional (side view) and top‐view SEM images of both the SD‐HCS/CNT@CoSe_2_ and M‐HCS/CNT@CoSe_2_ electrodes were obtained in the fresh state and after 200 cycles (Figure 6h–k and Figure S15). Initially, both electrodes had similar thickness (15.1 µm, Figure 6h, i). However, the SD‐HCS/CNT@CoSe_2_ electrode, synthesized via spray drying, exhibits a uniform distribution of particles with minimal gaps, suggesting higher tap density and more cohesive structure (Figure 6h). In contrast, the M‐HCS/CNT@CoSe_2_ electrode exhibits noticeable voids or gaps within the electrode matrix, likely owing to its non‐spray‐dried fabrication, where various particle sizes (ranging from bulk‐like clusters to nanoscale HCS) failed to form a homogeneous layer (Figure 6i). After 200 cycles, SD‐HCS/CNT@CoSe_2_ retained a shape and thickness largely similar to the fresh state, with only a slight increase to ∼16.2 µm, corresponding to a thickness expansion of 7.3% (Figure 6j). This minimal expansion reflects the ability of the uniformly CNT‐bound HCS to distribute the structural stress and buffer volumetric changes during repeated potassiation/depotassiation. In contrast, the M‐HCS/CNT@CoSe_2_ electrode's thickness grew from 15.1 to ∼24.4 µm, indicating a significant expansion of 50.6% (Figure 6k). Moreover, there is clear evidence of electrode collapse and crack formation, suggesting that locally weak regions in the electrode, where CNT and HCS are merely mixed rather than cohesively bound, cannot mitigate the stress induced by repeated charge–discharge cycles. This considerable deterioration is consistent with the abrupt performance drop observed for M‐HCS/CNT@CoSe_2_ after ∼65 cycles (Figure 6a). The top‐view SEM images confirm these observations. For SD‐HCS/CNT@CoSe_2_, the fresh electrode exhibited a uniform crack‐free surface (Figure S15a), and even after 200 cycles, only minor cracks appeared without major structural disruption (Figure S15b). In contrast, the M‐HCS/CNT@CoSe_2_ electrode started off with unevenly aggregated clusters embedded in the surface, leading to a rough and non‐uniform texture (Figure S15c). After 200 cycles, numerous voids emerged, presumably because of cluster detachment, and substantial electrode collapse occurred (Figure S15d). Overall, these results underscore the importance of spray drying in electrode design. A spray‐dried, CNT‐bound hollow carbon architecture not only enhances electrochemical performance but also preserves electrode integrity by effectively managing volumetric expansion during prolonged cycling.

The effect of varying the CoSe_2_ loading on potassium‐ion storage was investigated by synthesizing SD‐HCS/CNT@CoSe_2_ microclusters with nominal CoSe_2_ contents of 40, 60, and 80 wt.%, followed by a comparison of their CV profiles and cycling performance. As shown in Figure S16a, the 40‐wt.% sample exhibited discernible cathodic and anodic peaks associated with the CoSe_2_ conversion reaction; however, these peaks are noticeably weaker than those of the 60‐wt.% material (Figure 5a), which is a direct consequence of the less active material available for redox reactions. Correspondingly, the 40‐wt.% sample delivered a modest capacity of ∼264 mA h g^−1^, highlighting the limited capacity contribution from CoSe_2_ when its fraction was relatively low (Figure S16c). In contrast, the 80‐wt.% SD‐HCS/CNT@CoSe_2_ sample (Figure S16b) exhibits a pronounced CV profile that closely mirrors the characteristic CoSe_2_ peaks in the main sample (60 wt.%), but with an even sharper intensity owing to the larger amount of active material. Although this leads to a higher initial capacity, it also intensifies the volumetric expansion during charge–discharge (Figure S16c). The SEM images of the 80‐wt.% sample (Figure S17) reveals that CoSe_2_ agglomerates on the exterior of the SD‐HCS/CNT host, confirming that overload leads to phase separation and localized stress points. Collectively, these observations suggest that optimizing the CoSe_2_ loading is crucial for balancing high capacity with stable cycling. In this regard, the 60‐wt.% sample represents an effective balance, providing sufficient active material without compromising the electrode's structural stability.

The chemical composition of SEI formed on the SD‐HCS/CNT@CoSe_2_ electrode was meticulously investigated using depth profiling XPS and time‐of‐flight secondary ion mass spectrometry (TOF‐SIMS). In the initial C 1s and K 2p spectra (Figure 7a), the surface of the SD‐HCS/CNT@CoSe_2_ electrode exhibited organic components (C─O, C═O) alongside K─F and K─O peaks [48]. The subsequent disappearance of the K─O peak with increasing sputtering time suggests that the initial K─O signal originated from trace oxygen caused by partial surface oxidation. Notably, in the spectra obtained after 30 s of sputtering, the K─F peak was significantly intensified while the organic‐related peaks were attenuated. This trend indicates that the SEI interior is more enriched with inorganic components compared to its surface. This observation is further corroborated by the F 1s spectra (Figure 7b), where the K‐F and ‐SO_y_F signals, derived from the decomposition of the KFSI salt, are more pronounced in the bulk region than on the surface. This supports the formation of a stable, inorganic‐rich interphase within the SEI layer [49]. The O 1s spectra (Figure 7c) exhibited a consistent trend with the C 1s and K 2p results; the pristine surface showed K─O peaks along with C═O, ‐SO_y_F, and C‐O species. Among these, C═O and C─O are attributed to solvent decomposition, whereas ‐SO_y_F originates from the degradation of the KFSI salt [50]. The gradual weakening of organic‐related peaks toward the interior layers reinforces the structural consistency of the formed SEI.

**FIGURE 7:**
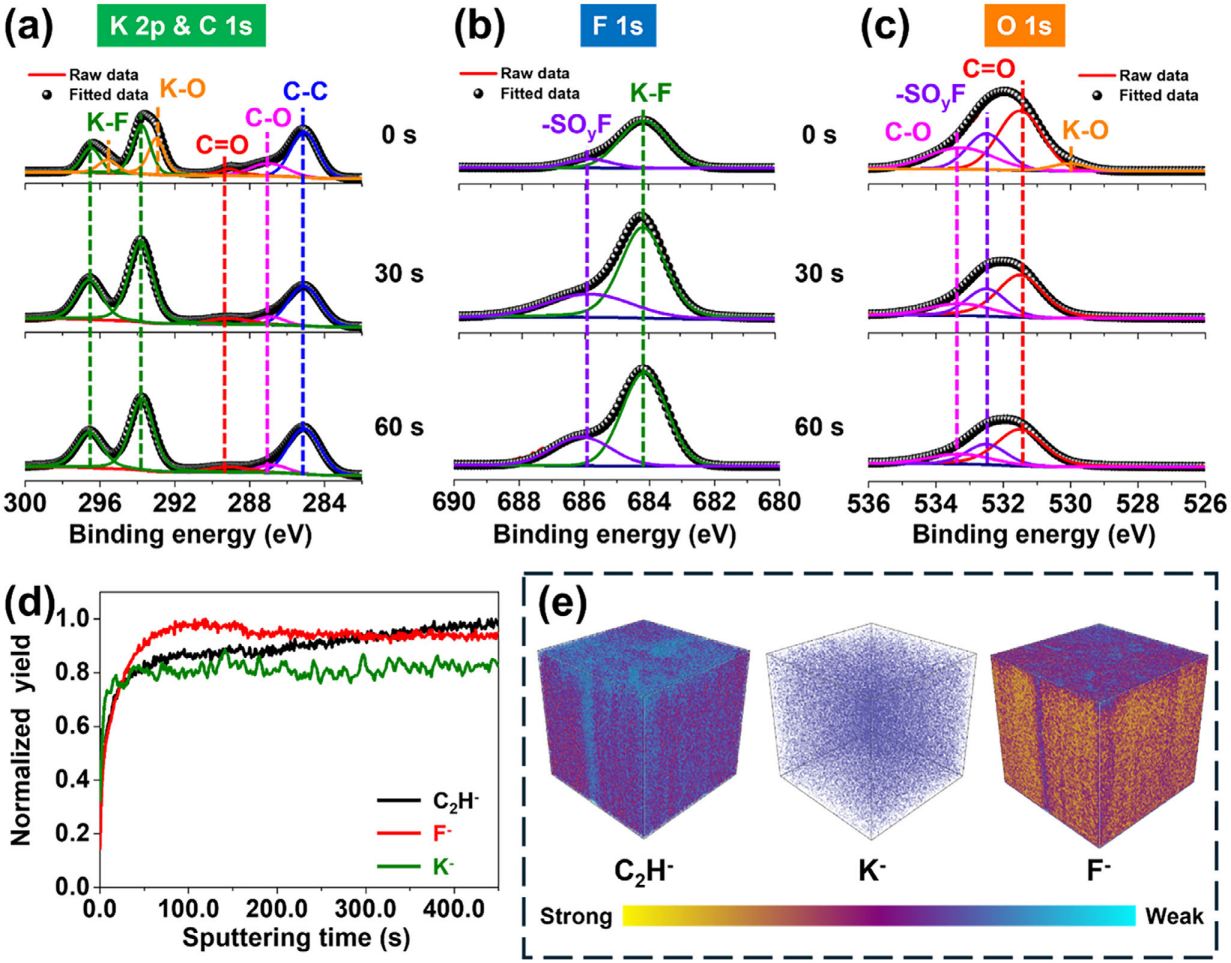
Ingredient analysis of the SEI on SD‐HCS/CNT@CoSe_2_ electrode. Depth profiling XPS of (a) K 2p and C 1s, (b) F 1s, and (c) O 1s spectra at different etching times. TOF‐SIMS characterization of the cycles SD‐HCS/CNT@CoSe_2_ electrode. (d) depth distribution profile measured from the surface in the bulk direction, and (e) 3D mapping result.

This compositional distribution was further validated through TOF‐SIMS analysis (Figure 7d,e). The depth‐dependent profiles were normalized based on the maximum intensity of each component from the surface toward the bulk direction. As shown in Figure 7d, the signals for all species exhibited a progressive increase with sputtering time. The corresponding 3D mapping results (Figure 7e) confirmed an abundant and uniform distribution of salt‐derived K‐ and F‐ ions throughout the SEI. While the organic‐based C_2_H‐ signal was relatively less intense, its ubiquitous presence suggests the successful formation of an organic‐inorganic hybrid SEI where organic species are uniformly interspersed within an inorganic‐dominant matrix.

In conclusion, the coexistence of K‐F and organic components across all layers confirms the formation of a robust organic‐inorganic hybrid SEI on the SD‐HCS/CNT@CoSe_2_ electrode. This hybrid architecture provides a synergistic effect, where the elasticity of the organic components effectively buffers the mechanical stress induced by volume expansion, while the high mechanical strength and ionic conductivity of the inorganic K─F component maintain interfacial stability. This synergy imparts superior structural durability compared to single‐component‐dominant SEIs, serving as a key factor for enhanced long‐term cycling stability [51].

To demonstrate its practical applicability, a full PIB cell was constructed (Figure 8a) using the SDHCS/CNT@CoSe_2_ as the anode and PB as the cathode (Figure S18). The voltage window of 0.7–4.0 V was selected to match the redox potential ranges of the SD‐HCS/CNT@CoSe_2_ anode and PB cathode (Figure 8b). SD‐HCS/CNT@CoSe_2_//PB exhibits high reversibility over prolonged cycling (Figure 8c), indicating closely matched redox activity between the two electrodes. Under this configuration, the SDHCS/CNT@CoSe_2_//PB cell delivered capacities of 300, 280, 267, 250, 230, 215, and 207 mA h g^−1^ at the current densities of 0.05, 0.1, 0.2, 0.4, 0.8, 1.0, and 2.0 A g^−1^, respectively (Figure 8d). When the rate was returned to 0.1 A g^−1^, the capacity recovered to 280 m Ah g^−1^, indicating excellent reversibility. In extended cycling at 0.2 A g^−1^, the full cell maintained ∼280 m Ah g^−1^ over 55 cycles with a CE close to 98% (Figure 8e). Finally, the practical utility of the SDHCS/CNT@CoSe_2_//PB cell was highlighted by powering a commercial LED, as shown in Figure 8f.

**FIGURE 8:**
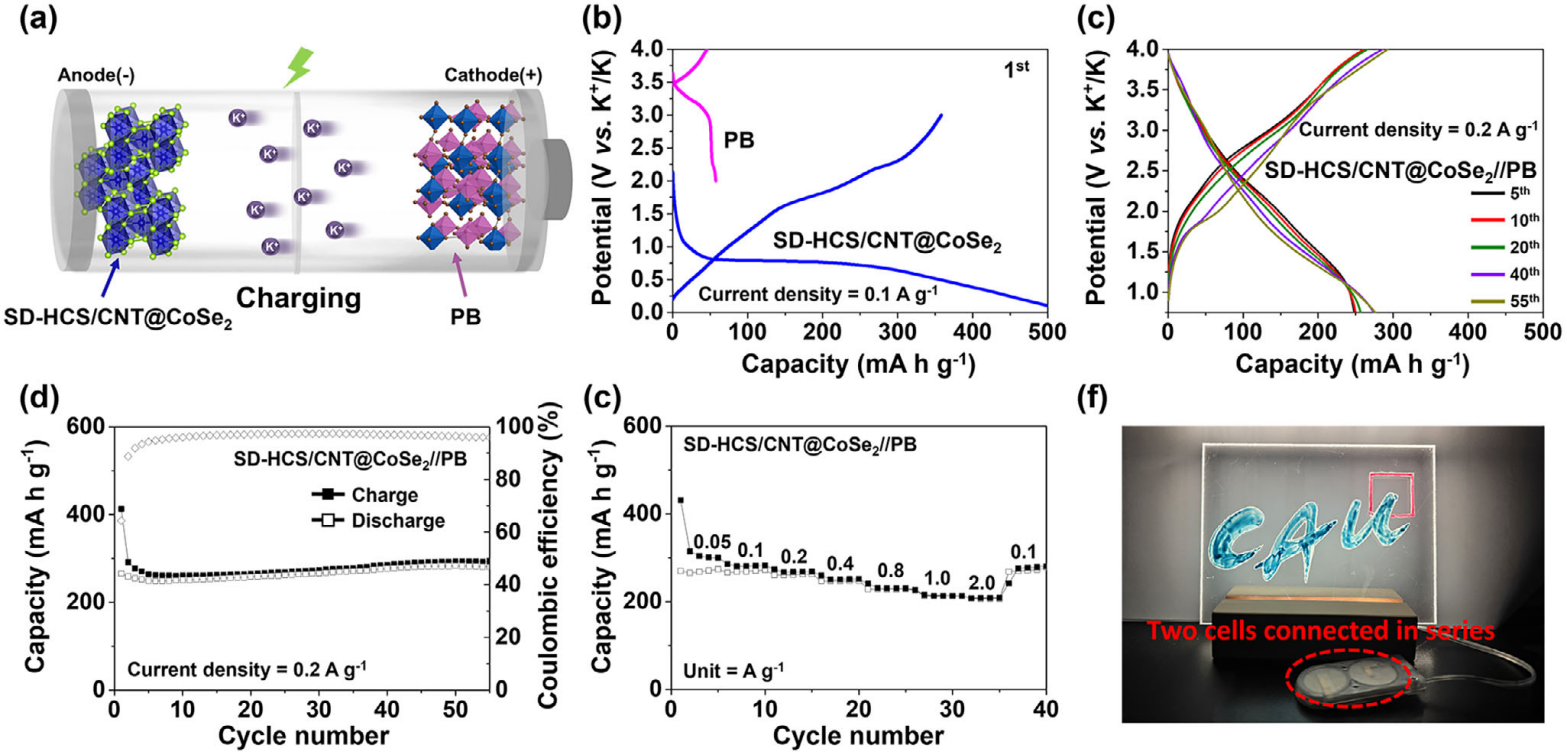
Full‐cell evaluation of SD‐HCS/CNT@CoSe_2_//PB potassium‐ion battery: (a) Schematic of full cell configuration using SD‐HCS/CNT@CoSe_2_ anode and PB cathode. (b) GCD profiles of SD‐HCS/CNT@CoSe_2_ anode and PB cathode in half cell at 0.1 A g^−1^. (c) GCD profiles at 0.2 A g^−1^. (c) Rate capability from 0.05 to 2.0 A g^−1^ with capacity recovery upon return to 0.1 A g^−1^. (d) Extended cycling at 0.2 A g^−1^ over 55 cycles. (e) Demonstration of full cell powering a commercial LED.

## 3 Conclusion

This paper proposes a strategy to stabilize the thermodynamically favored Co_3_​Se_4_​‐mediated conversion–insertion pathway in CoSe_2_​ via hierarchical, spray‐dried carbon architectures. Based on DFT, a Co_3_Se_4_‐mediated route (CoSe_2_ → Co_3_​Se_4_​ → Co_3_​KSe_4_​ → Co + K‐selenides) is energetically preferred to the conventional stepwise K‐insertion sequence and localizes the largest structural strain into a single, non‐potassiation step. Accordingly, this study designed SD‐HCS/CNT microclusters to confine CoSe_2_​ nanoparticles, provide a resilient electronic network, and buffer volume changes during the critical first transition. In situ X‐ray diffraction captured the disappearance of orthorhombic CoSe_2_ and the transient emergence of monoclinic Co_3_Se_4_, and the subsequent K‐containing phases, supporting the intermediate‐mediated conversion pathway predicted by DFT. Comparative structural and morphological analyses revealed that the spray‐drying process and CNT interconnectivity are essential for producing a mechanically cohesive, electronically percolating host that enables uniform precursor infiltration and controlled phase evolution. Collectively, the computational and experimental results confirm that stabilizing a latent intermediate (Co_3_Se_4_) through rational host design can mitigate the intrinsic issues of conversion‐type materials, improving reaction reversibility and structural integrity. In summary, thermodynamic pathway engineering combined with hierarchical mechanical and electronic confinement provides a generalizable design paradigm for advancing conversion‐type anodes in post‐lithium energy storage systems.

## Author Contributions

**Ho Rim Kim**: Writing – original draft, Visualization, Methodology, Investigation, Formal analysis, Data curation, Conceptualization. **Seohyeon Jang**: Writing – original draft, Visualization, Software, Methodology, Investigation, Formal analysis. **Hong Geun Oh**: Writing – review & editing, Visualization, Methodology, Investigation, Formal analysis. **Jaewoo Lee**: Writing – review & editing, Visualization, Investigation, Formal analysis. **Jihun Yeom**: Writing – review & editing, Visualization, Methodology, Investigation, Formal analysis. **Daiha Shin**: Visualization, Software, Resources, Investigation, Formal analysis. **Jiung Cho**: Writing – review & editing, Visualization, Software, Resources, Investigation. **Inho Nam**: Writing – review & editing, Supervision, Software, Resources, Project administration, Funding acquisition. **Seung‐Keun Park**: Writing – review & editing, Supervision, Software, Resources, Project administration, Funding acquisition, Conceptualization.

## Conflicts of Interest

The authors declare no conflicts of interest.

## Supporting information




**Supporting File**: advs76162‐sup‐0001‐SuppMat.docx.

## Data Availability

The data that support the findings of this study are available in the Supporting Information of this article.
