# Supplementary material for: Unveiling a Hidden Conversion Pathway in CoSe2 Anodes via Rationally Designed CNT‐Interwoven Hollow Carbon Microclusters for High‐Performance Potassium‐Ion Batteries
Source: Adv Sci (Weinh). 2026 Jun 22;13(55):e76162. doi: 10.1002/advs.76162 (PMC13336406; doi:10.1002/advs.76162)
Supplement: Supplementary file 1 [file ADVS-13-e76162-s001.docx]

**Supporting Information**

Unveiling a Hidden Conversion Pathway in CoSe_2_ Anodes via Rationally Designed CNT-Interwoven Hollow Carbon Microclusters for High-Performance Potassium-Ion Batteries

Ho Rim Kim^a‡^, Seohyeon Jang^a‡^, Hong Geun Oh^a^, Jaewoo Lee^a^, Jihun Yeom^a^, Daiha Shin^b^, Jiung Cho^c^, Inho Nam^a,d,e*^, and Seung-Keun Park^a,e*^

^a^Department of Intelligent Energy and Industry, Chung-Ang University, 84 Heukseok-ro, Dongjak-gu, Seoul 06974, Republic of Korea

^b^Metropolitan Seoul Center, Korea Basic Science Institute (KBSI), Seoul, Republic of Korea

^c^Department of Materials Science and Engineering, Hongik University, Sejong, Republic of Korea

^d^Department of Chemical Engineering, Chung-Ang University, 84 Heukseok-ro, Dongjak-gu, Seoul 06974, Republic of Korea

^e^Department of Advanced Materials Engineering, Chung-Ang University, 4726, Seodong-daero, Daedeok-myeon, Anseong, Gyeonggi-do 17546, Republic of Korea

^‡^These authors contributed equally to this work.

KEYWORDS: potassium-ion battery; hollow carbon sphere; cobalt selenides; spray drying; K-ion storage mechanism; carbon nanotubes.

**Characterization**

The morphologies and crystal structure of the synthesized samples were confined using analytic techniques, including Field emission-scanning electron microscopy (FE-SEM) (HITACHI S-5000), Field emission-transmission electron microscopy (FE-TEM) (JEM-2100F, Korea Basic Institute (Daegu)), X-ray diffraction (XRD) using Cu-Kα radiation, and X-ray photoelectron spectroscopy (XPS) (Thermos Fisher Scientific K-Alpha model). Information on the surface morphology and internal structure of the samples at the nanoscale was obtained through FE-SEM and FE-TEM analyses. XRD analysis revealed their crystal structure, and XPS analysis identified their chemical compositions. Surface areas and pore size distributions were determined using BET method with N_2_ as the adsorption gas. To determine the CoSe_2_ content in the samples, a Pyris 1 thermogravimetric (TG) analyzer (TA-SDT Q600) was utilized under air conditions, with a temperature range of 30–800 °C and a ramp rate of 10 °C·min^−1^. To determine the reaction mechanism that occurs in the CoSe_2_ composite, *in-situ* XRD analysis was performed using an X’Pert Pro MPD diffractometer (Malvern Panalytical) equipped with Cu Kα radiation (λ = 0.15418 nm) at the Korea Basic Science Institute (KBSI), Metropolitan Seoul Center (institutional instrument ID: GN002). The instrument was operated at an acceleration voltage of 40 kV and a current of 40 mA. A custom-designed in-situ cell was used for analysis, in which the SD-HCS/CNT@CoSe_2_ anode was cast onto a Be sheet, vacuum dried, and assembled inside an Ar-filled glove box. The charge-discharge profiles were recorded using a WPG100e potentiostat/galvanostat (WonATech, Republic of Korea) in galvanostatic mode with a current density of 20 mA g^-1^ within a voltage range of 0.01- 3.0 V. XRD measurements were conducted at room temperature to investigate the phase transition of the anode under different conditions.

**Computational Details**

First-principles calculations based on density functional theory (DFT) were performed using the Vienna Ab initio Simulation Package (VASP).^[1–4]^ The exchange–correlation interactions were treated using the Perdew–Burke–Ernzerhof (PBE) functional within the generalized gradient approximation (GGA). The interaction between ion cores and valence electrons was described using the projector augmented-wave (PAW) method.^[5–7]^ A kinetic energy cutoff of 400 eV was applied to the plane-wave basis set, and the electronic self-consistent field (SCF) convergence criterion was set to 10^-5^ eV. A minimum of 8 electronic SCF steps and 5 initial fixed-charge iterations were enforced to ensure numerical stability. The symmetry tolerance was set to 1.0 × 10^-6^ to maintain accurate space group recognition during relaxation.

For bulk Co, orthorhombic CoSe_2_, Co_3_Se_4_, Co_3_KSe_4_, CoK_x_Se_2_ (x = 1 – 3), K_2_Se, and K_2_Se_3_ structures, Gaussian smearing was used with a smearing width of 0.2 eV, and all structures were optimized until forces were below 0.03 eV/Å. The reference energy for potassium was obtained from bulk potassium metal in its body-centered cubic (bcc) structure (space group: Im-3m), which was optimized using the same computational settings. The resulting total energy per atom was used as the reference for potassium in the formation energy calculations.

Van der Waals interactions were considered using Grimme’s DFT-D3 method with zero damping.^[8]^ The Brillouin zone was sampled with Monkhorst–Pack k-point meshes such that the product of the lattice vector length and the number of k-points in each direction was maintained around 30 Å to ensure sufficient reciprocal-space sampling

The total and projected density of states (DOS and PDOS) were calculated using the self-consistent ground state wavefunctions. The Brillouin zone was sampled with 20×20×20 6×18×12 k-point mesh for CoSe_2_ and Co_3_Se_4_ bulk optimization to obtain well-resolved DOS features. For PDOS, atomic and orbital projections were carried out using LORBIT = 11, and the results were analyzed based on the DOSCAR output. To quantify the charge transfer between potassium (K) and the host lattice (Co–Se) during the potassiation process, Bader charge analysis was performed based on the grid-based decomposition of the electron density. The total electron density was partitioned into individual atomic volumes using the Henkelman group's Bader algorithm, ensuring a robust evaluation of the oxidation states. The charge difference (*ΔQ*) for each atom was determined by subtracting the calculated Bader charge (*Q_bader_*) from the number of valence electrons in the neutral atom (*Z_val_*), where *Z_val_* values of 9, 6, and 9 were used for Co (3*d*^8^ 4*s*^1^), Se (4*s*^2^ 4*p*^4^), and K (3*s*^2^ 3*p*^6^ 4*s*^1^), respectively. In this study, a positive ΔQ value indicates electron depletion (cationic character), whereas a negative ΔQ value indicates electron accumulation (anionic character), reflecting the extent of charge transfer and ionicity. For intermediate phases with multiple non-equivalent atomic sites (CoSe_2_, CoKSe_2_, Co_3_Se_4_ and Co_3_KSe_4_), the averaged charge values were utilized to represent the overall electronic environment of each element.

The formation energies for each reaction are defined as follows:

*E*_f_(1) = 1/3*E_bulk_*(Co_3_Se_4_) *+* 2/3*E_bulk_*(K_2_Se) *– E_bulk_*(CoSe_2_) *–* 4/3*E_atom_*(K)

*E*_f_(2) = 1/3*E_bulk_*(Co_3_KSe_4_) *–* 1/3*E_bulk_*(Co_3_Se_4_) *–* 1/3*E_atom_*(K)

*E*_f_(3) = *E_bulk_*(Co) *+* 1/3*E_bulk_*(K_2_Se) *+* 1/3*E_bulk_*(K_2_Se_3_) *–* 1/3*E_bulk_*(Co_3_KSe_4_) *– E_atom_*(K)

*E*_f_(4) = *E_bulk_*(CoKSe_2_) *– E_bulk_*(CoSe_2_) *– E_atom_*(K)

*E*_f_(5) = *E_bulk_*(CoK_2_Se_2_) *– E_bulk_*(CoSe_2_) *–* 2*E_atom_*(K)

*E*_f_(6) = *E_bulk_*(CoK_3_Se_2_) *– E_bulk_*(CoSe_2_) *–* 3*E_atom_*(K)

*E*_f_(7) = *E_bulk_*(Co) *+* 2*E_bulk_*(K_2_Se) *– E_bulk_*(CoSe_2_) *–* 4*E_atom_*(K)

The formation energies (E_f_) for reactions (1)–(7) were calculated using the total energies of the respective bulk phases at 0 K. In solid-state battery reactions, the internal energy change derived from DFT calculations is the dominant factor in determining the thermodynamic landscape. Although the system undergoes volume expansion during potassiation, the contribution of the PV term (pressure-volume work) at standard pressure is numerically negligible (< 10^-5^ eV) compared to the total energy change. Furthermore, the vibrational entropy contribution at room temperature is typically an order of magnitude smaller (< 0.1 eV) than the calculated internal energy changes, and thus it does not alter the overall reaction trends. Therefore, the 0 K formation energies were used as a reliable approximation for the Gibbs free energy. Additionally, the calculated E_f_ values were converted into theoretical average potentials (V vs. K^+^/K) to directly correlate the thermodynamic predictions with the electrochemical discharge profiles, according to the Nernst equation:

$$V= -\frac{E_{f}}{n}$$

, where n represents the number of electrons involved in each reaction step.

**Experiment**

**Synthesis of samples.** SD-HCS/CNTs were prepared using a simple spray drying process of a colloidal solution containing SiO_2_@resorcinol-formaldehyde (RF) and acid-treated CNTs, followed by carbonization and etching. SiO_2_@RF (precursor to HCS) was synthesized according to a previously reported procedure.^[9]^ Then, the resulting SiO_2_@RF powder was mixed with CNTs at a ratio of 2:1 (w/w), and the mixture was spray-dried under a flow rate of 5.3 mL min^−1^ and pressure of 2.0 bar. The inlet and outlet temperatures of the spray dryer were maintained at 220 °C and 100 °C, respectively. The as-obtained spray-dried powder was placed in an alumina boat and subjected to heat treatment at 900 °C for 3 h in an argon atmosphere to carbonize the RF shell. Subsequently, the internal SiO_2_ template was removed by etching in a 3 M NaOH aqueous solution to yield SD-HCS/CNT microclusters. For the metal selenide infiltration, 0.03 g of the SD-HCS/CNT microclusters was placed in a bowl, and 0.1 g of cobalt (II) nitrate hexahydrate was dissolved in 1 mL of ethanol. Then, 200 μL of this cobalt nitrate solution was sprayed onto the bowl three times, with intermediate drying steps after each spray to facilitate infiltration of cobalt nitrate into the SD-HCS/CNT host. Finally, the infiltrated sample was heated at 280 °C for 6 h in an H_2_/Ar atmosphere to form SD-HCS/CNT@CoSe_2_ microclusters.

**This file includes:**

- **Figure S1.** SEM images of spray-dried SiO_2_@RF/CNT microclusters at varying SiO_2_@RF:CNT mass ratios. (a, b) microclusters for ratios of 2:1, (c, d) 1:1, and (e, f) 1:2
- **Figure S2.** The hollow nature of the HCS was confirmed by SEM images showing partially broken shells within the microclusters, clearly revealing their empty interior.
- **Figure S3.** TEM characterization of SD-HCS/CNT microclusters. (a) Low-magnification image showing hollow carbon spheres with internal diameters of ~300 nm. (b) Medium-magnification view illustrating CNT filaments interconnecting adjacent HCS within the cluster. (c) High-magnification image of a single HCS revealing a uniform carbon shell thickness of ~30 nm. (d) Ultra-high-magnification detail showing mesopores (~10 nm) and voids within the carbon shell.
- **Figure S4.** SEM images of M-HCS/CNT microclusters.
- **Figure S5.** SEM images of dextrin‐assembled SD‐HCS (a, b) and CNT‐reinforced SD‐HCS/CNT (c, d) after 15 min ball‐milling
- **Figure S6.** Nitrogen adsorption-desorption analysis of HCS and SD-HCS/CNT. (a) N_2_ adsorption-desorption isotherm for pristine HCS. (b) N_2_ adsorption–desorption isotherm for SD-HCS/CNT.
- **Figure S7.** Morphology and cycling performance of SD-HCS/CNT@CoSe_2_ prepared with different HCS:CNT ratios. (a, b) SEM images showing CoSe_2_ nanoparticles deposited on the outer surface of microclusters for HCS:CNT = 1:1 and 1:2. (c) Cycling stability of SD-HCS/CNT@CoSe_2_ electrodes at various HCS:CNT ratios (0.1 A g^-1^, 0.5 A g^-1^)
- **Figure S8.** XPS analysis of the SD-HCS/CNT@CoSe_2_ (a) full survey spectrum, (b) high-resolution C 1s spectrum, (c) Co 2p spectrum, (d) Se 3d spectrum.
- **Figure S9.** In-situ XRD intensity map of the monoclinic Co_3_Se_4_ (31-1) reflection during the second K^+^ discharge-charge cycle of SD-HCS/CNT@CoSe_2_
- **Figure S10.** (a) Theoretical voltage profiles derived from DFT calculations for the first and subsequent discharge cycles. (b) Three-cycle in-situ XRD contour plot for SD-HCS/CNT@CoSe_2_ during K^+^ discharge-charge (0.01-3 V). The arrows and shaded regions illustrate the correlation between the calculated potential plateaus for potassiation/decomposition reactions and the corresponding experimental phase evolution.
- **Figure S11.** TEM images of SD-HCS/CNT@CoSe_2_ microclusters (a, b) after full discharge to 0.01 V, (c, d) after full charge to 3.0 V.
- **Figure S12** CV curves at 0.1 mV s^-1^ (a) M-HCS/CNT@CoSe_2_, (b) SD-HCS/CNT. (c) CV curves at varying scan rates (0.1–0.8 mV s^-1^) for capacitive contribution analysis. (d) Capacitive fraction vs. scan rate for M-HCS/CNT@CoSe_2_ electrodes.
- **Figure S13** Current step diagram at 3.0V K^+^/K for parameter determination
- **Figure S14** Half-cell electrochemical setup and initial performance of SD-HCS/CNT@CoSe_2_, M-HCS/CNT@CoSe_2_ and SD-HCS/CNT.
- **Figure S15** Top-view SEM images of SD-HCS/CNT@CoSe_2_ and M-HCS/CNT@CoSe_2_ electrodes before and after 200 cycles (a, c) Top-view of fresh electrode surfaces. (b, d) Top-view after 200 cycles.
- **Figure S16** CV curves of SD-HCS/CNT@CoSe₂ microclusters with different CoSe_2_ loadings (a) 40 wt %, and (b) 80 wt %. (c) and Cycle performance of SD-HCS/CNT@CoSe₂ microclusters with different CoSe_2_ loadings (40, 60, and 80 wt %).
- **Figure S17** SEM images of SD-HCS/CNT@CoSe_2_ with 80 wt % CoSe_2_ loading (a) low-magnification view, (b) high-magnification view.
- **Figure S18** SEM image of prussian blue (PB) (a) low magnification, (b) high magnification.
- **Table S1.** Bader charge analysis of intermediate phases during potassiation.
- **Table S2.** Textural parameters of HCS and SD-HCS/CNT hosts obtained from nitrogen adsorption-desorption isotherms, including BET surface area, total pore volume, and average pore diameter.
- **Table S3.** Comparison table with previously reported data and our work

**Figure S1.** SEM images of spray-dried SiO_2_@RF/CNT microclusters at varying SiO_2_@RF:CNT mass ratios. (a, b) microclusters for ratios of 2:1, (c, d) 1:1, and (e, f) 1:2

**Figure S2.** The hollow nature of the HCS was confirmed by SEM images showing partially broken shells within the microclusters, clearly revealing their empty interior.

**Figure S3.** TEM characterization of SD-HCS/CNT microclusters. (a) Low-magnification image showing hollow carbon spheres with internal diameters of ~300 nm. (b) Medium-magnification view illustrating CNT filaments interconnecting adjacent HCS within the cluster. (c) High-magnification image of a single HCS revealing a uniform carbon shell thickness of ~30 nm. (d) Ultra-high-magnification detail showing mesopores (~10 nm) and voids within the carbon shell.

**Figure S4.** SEM images of M-HCS/CNT microclusters.

**Figure S5.** SEM images of dextrin‐assembled SD‐HCS (a, b) and CNT‐reinforced SD‐HCS/CNT (c, d) after 15 min ball‐milling

**Figure S6.** Nitrogen adsorption-desorption analysis of HCS and SD-HCS/CNT. (a) N_2_ adsorption-desorption isotherm for pristine HCS. (b) N_2_ adsorption–desorption isotherm for SD-HCS/CNT.

**Figure S7.** Morphology and cycling performance of SD-HCS/CNT@CoSe_2_ prepared with different HCS:CNT ratios. (a, b) SEM images showing CoSe_2_ nanoparticles deposited on the outer surface of microclusters for HCS:CNT = 1:1 and 1:2. (c) Cycling stability of SD-HCS/CNT@CoSe_2_ electrodes at various HCS:CNT ratios (0.1 A g^-1^, 0.5 A g^-1^)

**Figure S8.** XPS analysis of the SD-HCS/CNT@CoSe_2_ (a) full survey spectrum, (b) high-resolution C 1s spectrum, (c) Co 2p spectrum, (d) Se 3d spectrum.

**Figure S9.** In-situ XRD intensity map of the monoclinic Co_3_Se_4_ (31-1) reflection during the second K^+^ discharge-charge cycle of SD-HCS/CNT@CoSe_2_

**Figure S10.** (a) Theoretical voltage profiles derived from DFT calculations for the first and subsequent discharge cycles. (b) Three-cycle in-situ XRD contour plot for SD-HCS/CNT@CoSe_2_ during K^+^ discharge-charge (0.01-3 V). The arrows and shaded regions illustrate the correlation between the calculated potential plateaus for potassiation/decomposition reactions and the corresponding experimental phase evolution.

**Figure S11.** TEM images of SD-HCS/CNT@CoSe_2_ microclusters (a, b) after full discharge to 0.01 V, (c, d) after full charge to 3.0 V.

**Figure S12.** CV curves at 0.1 mV s^-1^ (a) M-HCS/CNT@CoSe_2_, (b) SD-HCS/CNT. (c) CV curves at varying scan rates (0.1–0.8 mV s^-1^) for capacitive contribution analysis. (d) Capacitive fraction vs. scan rate for M-HCS/CNT@CoSe_2_ electrodes.

**Figure S13.** Current step diagram at 3.0V K^+^/K for parameter determination

**Figure S14.** Half-cell electrochemical setup and initial performance of SD-HCS/CNT@CoSe_2_, M-HCS/CNT@CoSe_2_ and SD-HCS/CNT. (a) GCD profiles at 0.1 A g^-1^ (b) GCD profiles at selected cycles of M-HCS/CNT@CoSe_2_

**Figure S15.** top-view SEM images of SD-HCS/CNT@CoSe_2_ and M-HCS/CNT@CoSe_2_ electrodes before and after 200 cycles (a, c) Top-view of fresh electrode surfaces, (b, d) Top-view after 200 cycles.

**Figure S16.** CV curves of SD-HCS/CNT@CoSe₂ microclusters with different CoSe_2_ loadings (a) 40 wt %, and (b) 80 wt %. (c) and Cycle performance of SD-HCS/CNT@CoSe_2_ microclusters with different CoSe_2_ loadings (40, 60, and 80 wt %).

**Figure S17.** SEM images of SD-HCS/CNT@CoSe_2_ with 80 wt % CoSe_2_ loading (a) low-magnification view, (b) high-magnification view.

**Figure S18.** SEM image of Prussian blue (PB) (a) low magnification, (b) high magnification.

**Table S1.** Bader charge analysis of intermediate phases during potassiation.

|  | **CoSe_2_ → CoKSe_2_** | **Co_3_Se_4_ → Co_3_KSe_4_** |
| --- | --- | --- |
| K | + 0.49 *e^-^* | + 0.75 *e^-^* |
| Se | – 0.23 *e^-^* → – 0.40 *e^-^* (Δ 0.17 *e^-^*) | – 0.36 *e^-^* → – 0.52 *e^-^* (Δ 0.16 *e^-^*) |
| Co | + 0.46 *e^-^* → + 0.32 *e^-^* (Δ 0.14 *e^-^*) | + 0.48 *e^-^* → + 0.44 *e^-^* (Δ 0.04 *e^-^*) |

| **Material** | **BET (m^2^ g^-1^)** | **Total pore volume** | **Average pore  diameter** |
| --- | --- | --- | --- |
| HCS | 509.46 | 1.34 | 17.65 |
| SD-HCS/CNT (HCS:CNT = 1:2) | 251.37 | 1.36 | 20.14 |
| SD-HCS/CNT (HCS:CNT = 1:1) | 358.39 | 1.91 | 19.58 |
| SD-HCS/CNT (HCS:CNT = 2:1) | 608 | 2.93 | 17.25 |

**Table S2.** Textural parameters of HCS and SD-HCS/CNT hosts obtained from nitrogen adsorption-desorption isotherms, including BET surface area, total pore volume, and average pore diameter.

**Table S3.** Comparison table with previously reported data and our work

| **Material** | **Voltage window [V]** | **Current density**  **[A g^-1^]** | | **Discharge capacity**  **[mA h g^-1^]** | **Cycle** | **Rate performance**  **[mA h g^-1^]**  **(Current density)** | **Ref.** | | |
| --- | --- | --- | --- | --- | --- | --- | --- | --- | --- |
| SD-HCS/CNT@CoSe_2_ | 0.01-3.0 | 0.5 | 370 | | 200 | 264 (2.0 A g^-1^) | | Our work |  |
| Co_0.8_Se@C | 0.001-3.0 | 0.4 | 299 | | 400 | 483 (5.0 A g^-1^) | | ^[10]^ |  |
| CoSe_2_@C-1:1 | 0.01-3.0 | 0.1 | 366.1 | | 100 | 281 (5.0 A g^-1^) | | ^[11]^ |  |
| CoSe_2_/VSe_2_@NCNF | 0.01-3.0 | 1 | 400 | | 200 | 325 (5.0 A g^-1^) | | ^[12]^ |  |
| CoSe_2_⊂SPNC⊂rGO | 0.01-3.0 | 0.5 | 208.8 | | 500 | 115 (1.5 A g^-1^) | | ^[13]^ |  |
| CoSe_2_-NPC@CNS | 0.01-3.0 | 0.1 | 320.8 | | 100 | 188.8 (5 A g^-1^) | | ^[14]^ |  |
| CoSe_2_–FeSe_2_@C-II | 0.01-2.2 | 0.1 | 420 | | 100 | 275 (2.0 A g^-1^) | | ^[15]^ |  |
| CoMoSe@NCP/NCFs | 0.01-3.0 | 2 | 206 | | 2500 | 161 (10 A g^-1^) | | ^[16]^ |  |
| CoSe_2_@N-rGO | 0.01-3.0 | 0.2 | 421 | | 100 | 199.3 (1.0 A g^-1^) | | ^[17]^ |  |
| 3D MoSe_2_/CoSe_2_@NPC | 0.01-3.0 | 0.1 | 237 | | 200 | 215.6 (3.2 A g^-1^) | | ^[18]^ |  |
| ZnSe/CoSe_2_@C |  | 0.3 | 369 | | 90 | 180 (1.5 A g^-1^) | | ^[19]^ |  |

**References**

[1]	G. Kresse, J. Furthmüller, “Efficient iterative schemes for ab initio total-energy calculations using a plane-wave basis set” *Phys. Rev. B* **1996**, *54*, 11169.

[2]	G. Kresse, J. Furthmüller, “Efficiency of ab-initio total energy calculations for metals and semiconductors using a plane-wave basis set” *Comput. Mater. Sci.* **1996**, *6*, 15–50.

[3]	G. Kresse, J. Hafner, “Ab initio molecular-dynamics simulation of the liquid-metal–amorphous-semiconductor transition in germanium” *Phys. Rev. B* **1994**, *49*, 14251.

[4]	G. Kresse, J. Hafner, “Ab initio molecular dynamics for liquid metals” *Phys. Rev. B* **1993**, *47*, 558.

[5]	P. Pulay, “Convergence acceleration of iterative sequences. The case of SCF iteration” *Chem. Phys. Lett.* **1980**, *73*, 393–398.

[6]	P. E. Blöchl, “Projector augmented-wave method” *Phys. Rev. B* **1994**, *50*, 17953.

[7]	J. P. Perdew, K. Burke, M. Ernzerhof, “Generalized gradient approximation made simple” *Phys. Rev. Lett.* **1996**, *77*, 3865.

[8]	S. Grimme, J. Antony, S. Ehrlich, H. Krieg, “A consistent and accurate ab initio parametrization of density functional dispersion correction (DFT-D) for the 94 elements H-Pu” *J. Chem. Phys.* **2010**, *132*.

[9]	J.-S. Park, S. Y. Yang, J.-K. Lee, Y. C. Kang, “A novel strategy for encapsulating metal sulfide nanoparticles inside hollow carbon nanosphere-aggregated microspheres for efficient potassium ion storage” *J. Mater. Chem. A* **2022**, *10*, 17790–17800.

[10]	C. A. Etogo, H. Huang, H. Hong, G. Liu, L. Zhang, “Metal–organic-frameworks-engaged formation of Co_0. 85_Se@ C nanoboxes embedded in carbon nanofibers film for enhanced potassium-ion storage” *Energy Storage Mater.* **2020**, *24*, 167–176.

[11]	G. Yang, C. Yan, P. Hu, Q. Fu, H. Zhao, Y. Lei, “Synthesis of CoSe_2_ reinforced nitrogen-doped carbon composites as advanced anodes for potassium-ion batteries” *Inorg. Chem. Front.* **2022**, *9*, 3719–3727.

[12]	S. Xiao, Y. Zhu, X. Liu, R. Zhang, J. Qin, H. Chen, X. Niu, J. Wang, J. Jiang, J. S. Chen, “Promoting fast potassium storage in CoSe_2_/VSe_2_ non-layered/layered heterostructured nanofibers” *J. Mater. Chem. A* **2024**, *12*, 30289–30297.

[13]	Z. Zhao, C. Gao, J. Fan, P. Shi, Q. Xu, Y. Min, “Dual confinement of CoSe_2_ nanorods with polyphosphazene-derived heteroatom-doped carbon and reduced graphene oxide for potassium-ion batteries” *ACS Omega* **2021**, *6*, 17113–17125.

[14]	Q. Liu, X. Tan, X. Li, Y. Li, X. Han, S. Cui, D. Xu, Y. Liu, R. Wang, Q. Zhao, “Flower-like CoSe_2_/N, P-doped carbon microspheres accommodated on carbon nanosheets for high-performance potassium-ion batteries” *J. Energy Storage* **2024**, *87*, 111449.

[15]	H. Shan, J. Qin, Y. Ding, H. M. K. Sari, X. Song, W. Liu, Y. Hao, J. Wang, C. Xie, J. Zhang, “Controllable heterojunctions with a semicoherent phase boundary boosting the potassium storage of CoSe_2_/FeSe_2_” *Adv. Mater.* **2021**, *33*, 2102471.

[16]	Z. Ming Yu, J. Hui Jia, G. Yong Wang, Z. Wen, C. Cheng Yang, Q. Jiang, “Confining CoSe/MoSe_2_ Heterostructures in Interconnected Carbon Polyhedrons for Superior Potassium Storage” *ChemSusChem* **2025**, *18*, e202402434.

[17]	H. Zheng, H.-S. Xu, J. Hu, H. Liu, L. Wei, S. Wu, J. Li, Y. Huang, K. Tang, “Electrochemical performance of CoS_2_ with mixed phases decorated with N-doped rGO in potassium-ion batteries” *RSC Adv.* **2022**, *12*, 21374–21384.

[18]	Y. Ma, X. Li, L. Li, Y. Zhang, W. Zhang, “Constructing MoSe_2_/CoSe_2_ bimetallic selenides distributed on n-doped porous carbon matrix for high-efficiency potassium-ion storage” *Colloids Surf. Physicochem. Eng. Asp.* **2025**, *719*, 137021.

[19]	S. Iqbal, L. Wang, Z. Kong, Y. Zhai, F. Wang, Z. Jing, X. Sun, B. Wang, X. He, J. Dou, “2D Se‐Rich ZnSe/CoSe_2_@ C Heterostructured Composite as Ultrastable Anodes for Alkaline‐Ion Batteries” *Small* **2024**, *20*, 2404193.
